# Branching principles of animal and plant networks identified by combining extensive data, machine learning and modelling

**DOI:** 10.1098/rsif.2020.0624

**Published:** 2021-01-06

**Authors:** Alexander B. Brummer, Panagiotis Lymperopoulos, Jocelyn Shen, Elif Tekin, Lisa P. Bentley, Vanessa Buzzard, Andrew Gray, Imma Oliveras, Brian J. Enquist, Van M. Savage

**Affiliations:** 1 Institute for Quantitative and Computational Biology, University of California, Los Angeles, CA, USA; 2 Department of Ecology and Evolutionary Biology, University of California, Los Angeles, CA, USA; 3 Department of Computational Medicine, David Geffen School of Medicine, University of California, Los Angeles, CA, USA; 4 Department of Electrical Engineering and Computer Science, Massachusetts Institute of Technology, Cambridge, MA, USA; 5 Department of Biology, Sonoma State University, Rohnert Park, CA, USA; 6 Department of Ecology and Evolutionary Biology, University of Arizona, Tucson, AZ, USA; 7 School of Natural Resources and the Environment, University of Arizona, Tucson, AZ, USA; 8 Environmental Change Institute, School of Geography and the Environment, University of Oxford, Oxford, UK; 9 Santa Fe Institute, Santa Fe, NM, USA; 10 Department of Computer Science, Tufts University, Medford, MA, USA

**Keywords:** metabolic scaling, vascular biology, branching networks, machine learning

## Abstract

Branching in vascular networks and in overall organismic form is one of the most common and ancient features of multicellular plants, fungi and animals. By combining machine-learning techniques with new theory that relates vascular form to metabolic function, we enable novel classification of diverse branching networks—mouse lung, human head and torso, angiosperm and gymnosperm plants. We find that ratios of limb radii—which dictate essential biologic functions related to resource transport and supply—are best at distinguishing branching networks. We also show how variation in vascular and branching geometry persists despite observing a convergent relationship across organisms for how metabolic rate depends on body mass.

## 1. Introduction

**Figure 1.**
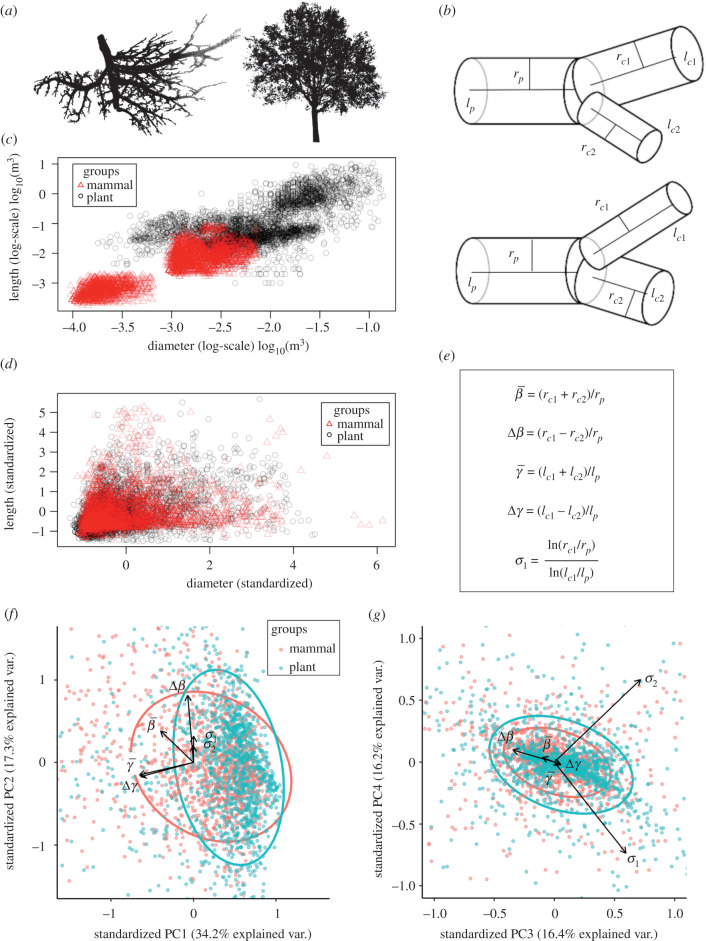
(*a*) Examples of mouse lung and angiosperm branching networks. (*b*) Diagrams of *positive* (top) and *negative* (bottom) asymmetric branching. (*c*) Scatter plot of lengths and diameters of all data studied, logarithmically scaled, shows trivial size-based clustering. (*d*) Scatter plot of standardized (zero mean and unit variance) lengths and diameters of all data studied shows non-informative overlap. (*e*) Definitions of biophysically motivated transformations: average and difference radial scale factors ($\bar{\beta },\Delta \beta$) related to hydraulic resistance, length-scale factors ($\bar{\gamma },\Delta \gamma$) related to space-filling and sibling slenderness scaling exponents (*σ*_1_, *σ*_2_) related to gravitational bending and buckling. (*f*,*g*) First through fourth principal components of variables defined in (*e*), ellipses are contours of 75% quantiles for bivariate principal components, and vector lengths indicate PC loadings. (*f*) PC1 and PC2 show large extent of variance associated with radial and length-scale factors, with group clustering determined separately by Δ*β* for plants and $\bar{\beta }$ for mammals (figure 3). (*g*) PC3 and PC4 show variances due to asymmetric radial scaling (Δ*β*) and linear combinations of sibling slenderness scaling exponents.

It is a great challenge to decipher which features of biological branching networks are shared, which are different, and when these differences matter [1,2]. For instance, branching in plant and animal networks exhibits strikingly similar features despite profound physiological and environmental differences (e.g. carbon dioxide and sap versus oxygen and blood, mobile versus stationary organisms, heart and pulsatile flow versus non-pulsatile flow) [3–11]. Similarly, differences in loopiness and ‘noisiness’ are well documented between vascular branching in tumours or stroke-damaged tissue versus healthy tissue [2,12,13]. The shared branching features are argued to lead to functional convergence in plant and animal networks via biological rates despite the notable physiological differences just listed [14–16]. Yet, the extent of shared versus distinct branching features has not been systematically and quantitatively analysed across plants and animals in the same study. Consequently, there is a need to understand the forces that shape the full spectrum of form and function in branching networks (figure 1*a*).

The classification of branching architectures is historically based on coarse qualitative differences in morphological features. Examples include: classifying lobes of the liver based on independent blood supply [17]; or the paired/un-paired ordering of plant leaves along a stem [18]. Recent efforts have identified gene expression profiles related to branching phenotypes, with examples of branching in the developing lung as being planar versus tetrahedral in orientation [19], or branching in the developing kidney based on the number of terminal vessels downstream from (or distal to) two sibling branches [20]. However, these empirically motivated classifications still fall short of relating patterns in vascular form to biophysical and biomechanical function.

With recent advances in automated methods of image analysis developed by us and others [4,5,21], increasing amounts of data are becoming available to tackle these problems. The tools that are missing are efficient and accurate algorithms for categorizing branching across whole networks and different organisms. In this paper, we apply machine learning methods to theoretically informed feature spaces to leverage all available information and technology to achieve these goals.

We analyse the largest-ever compilation of branching network data, with over 58 distinct networks and approximately 8000 vessels or tree limbs (table 1).

**Table 1.** Basic measures of the different vascular branching networks studied.

vascular branching network	trunk radius (cm)	trunk length (cm)	mean tip radius (mm)	mean tip length (mm)	number of tips	number of generations	number of junctions	total number of vessels
ML (*N* = 1)	0.0686	0.103	0.098 (0.055)	0.709 (0.434)	688	9	660	1348
HHT (*N* = 18)	0.383 (0.09)	2.77 (1.74)	0.855 (0.474)	6.950 (7.18)	50 (30)	6 (1)	48 (30)	1891
Balsa (*N* = 1)	18.8	1170	5.97 (4.99)	125 (151)	357	8	292	649
Piñon (*N* = 1)	5.73	5.4	2.30 (1.61)	24.5 (18.2)	1286	10	813	2099
Ponderosa (*N* = 5)	2.06 (0.769)	30.6 (30.1)	2.25 (0.714)	77.8 (62.0)	31 (21)	5 (1)	23 (21)	312
roots (*N* = 314)	0.307 (0.293)	25.9 (23.8)	1.19 (0.838)	89.2 (74.1)	2 (2)	1 (1)	1 (1)	1231
AS/GS tips (*N* = 31)	0.320 (0.103)	10.8 (6.3)	0.516 (0.318)	57.8 (53.7)	15 (11)	4 (1)	12 (9)	914

Physical dimensions and counts of various network properties, including: initial (trunk) and terminal (tip) vessels and branches. For single network datasets (*N* = 1) reported values are exact. For multi-network datasets (*N* > 1), values are averages with standard deviations reported in parentheses. For a given network, the number of generations, $N_{\mathrm{GEN}}$, is determined from the number of tips, $N_{TIPS}$, as $N_{\mathrm{GEN}}=\ln(N_{TIPS})/\ln(2)$, and rounded to the nearest integer. Due to approximate log-normality of distributions, means and standard deviations were determined in log-space and back transformed.

We collected these data over the last decade for both mammalian cardiovascular systems and plant architecture in both angiosperms and gymnosperms. Two mammalian networks are studied, the first being the major arterial branching junctions of the human head and torso (HHT) for 18 adult individuals (*H. sapiens*) collected using contrast-enhanced magnetic resonance angiography on a 3 T Siemens Trio scanner with voxel dimensions between 700 × 700 × 800 μm^3^ and 800 × 800 × 900 μm^3^ [5]. The second mammalian network is the full pulmonary vascular branching of one wild-type adult mouse lung (ML) (*M. musculus*) collected using a combination of vascular casting with MICROFIL and micro computed tomography on a *μ*CT 40, ScanCo Medical scanner with 10 μm isotropic voxel spacing [9]. All mammalian network data were acquired using the open source software Angicart [22].

The plant networks consist of: (i) whole, above-ground, adult trees for one Balsa (*O. pyramidale*), one Piñon (*P. edulis*) and five Ponderosa pines (*P. ponderosa*) [3], (ii) an array of angiosperm root clusters belonging to Andean tropical montane cloud forests [23] and (iii) a collection of 50 cm long clippings of the terminal ends of canopy branches from three species each of angiosperms (AS Tips) and gymnosperms (GS Tips) comprised of Maple (*A. grandidentatum*), Scrub Oak (*Q. gambelii*), Robinia (*R. neomexicana*), White Fir (*A. concolor*), Douglas Fir (*P. menziesii*) and White Pine (*P. strobiformis*). Tree measurements—all done destructively by hand—are of the external branching structures (limbs), not the xylem that are directly responsible for water transport. Scaling relationships for the external limbs directly determine similar relationships for the internal xylem based on previous empirical studies [24,25] and established branching theory [11,26], thus enabling comparisons of plant and animal networks for the structure, flow and function in the present study [3,21,27].

To search for patterns, machine learning is often applied to the full set of untransformed, standardized raw data. This is done because (i) in the absence of a prior theory, it is the most straightforward approach; and (ii) some practitioners of machine learning prefer to have a model- or theory-agnostic method arguably free of bias. One aim of this work is to examine and contrast results from theory-informed approaches with those that are theory-free.

While the raw data represent one feature space, there are always infinitely more choices of feature spaces based on specific combinations, subsets, mathematical operations (e.g. logarithms or ratios), or other transformations of the raw data (figure 1*c*,*d*,*e*). Informed choices of feature space hold the promise of greatly improving the convergence time, accuracy and inference of machine learning algorithms. Here, we show how crucial this choice can be and the roles that our understanding of the underlying biology can play in its selection. We further demonstrate that this approach identifies key strengths and weaknesses in the theory used to guide the transformations, and thus informs our understanding, or lack thereof, of the underlying biology and physics.

The default choice for feature spaces for our networks would be the centred and standardized raw data—all vessel radii and lengths for branching networks. However, theory grounded in evolution, biology and physics predicts that the parent-to-child ratios of radii and length—along with associated scaling exponents throughout the networks [10,11]—encapsulate the most biologically informative properties because they are directly tied to organismic function. Specifically, numerous models tie these ratios to the ability of branching networks to efficiently fill space and to deliver resources [7,10,11,26,28]. The fine-scale relationships between fluid flow, global vascular or branching architecture and vessel or branch morphology are indeed complex [29]. Despite this, much information can be gleaned from the connections between the radial scale factors and hydrodynamics and the length-scale factors and space-filling as first-order effects [1,3,5,9]. As candidates for second-order effects, we also examine branch slenderness exponents. These couple the radial and length-scale factors and inform the likelihood that a branch will experience gravitational buckling under its own weight [6,27,30].

We use recent theory developed by some of us (Brummer *et al.* [28]) for the asymmetric branching patterns that are pervasive throughout our data. In this theory, the two sibling vessels (labelled *c*1 and *c*2, figure 1*b*) and the parent vessel (labelled *p*) are combined to give two radial scale factors *β*_1_ = *r*_*c*1_/*r*_*p*_ and *β*_2_ = *r*_*c*2_/*r*_*p*_ and two length-scale factors *γ*_1_ = *l*_*c*1_/*l*_*p*_ and *γ*_2_ = *l*_*c*2_/*l*_*p*_. Thus the *average* radial and length-scale factors are

1.1 $$\bar{\beta }=\frac{\beta_{1}+\beta_{2}}{2}\;\text{and}\;\bar{\gamma }=\frac{\gamma_{1}+\gamma_{2}}{2}.$$

To capture sibling branch asymmetry the *difference* radial and length-scale factors are

1.2 $$\Delta \beta =\frac{\beta_{1}-\beta_{2}}{2}\;\mathrm{and}\;\;\;\;\Delta \gamma =\frac{\gamma_{1}-\gamma_{2}}{2}.$$

Corresponding constraint equations for area-preserving and space-filling branching—used in canonical optimization models—are

1.3 $${\left(\bar{\beta }+\Delta \beta \right)}^{2}+{\left(\bar{\beta }-\Delta \beta \right)}^{2}=1$$

and

1.4 $${\left(\bar{\gamma }+\Delta \gamma \right)}^{3}+{\left(\bar{\gamma }-\Delta \gamma \right)}^{3}=1.$$

Separately, susceptibility to gravitational buckling is quantified in the slenderness exponents [30], which relate the scaling of radii to the scaling of lengths as

1.5 $$\sigma_{1}=\frac{\ln(r_{c1}/r_{\;p})}{\ln(l_{c1}/l_{\;p})}\;\text{and}\;\sigma_{2}=\frac{\ln(r_{c2}/r_{\;p})}{\ln(l_{c2}/l_{\;p})}.$$

It is not *a priori* obvious which combinations of the scale factors will work best as a feature space for discriminating vascular networks. If dynamics of blood flow dominate the formation and evolution of vascular architecture, then variation in scale factors involving vessel radius would be expected to be most informative because vascular theory and empirical evidence show blood flow is most strongly determined by vessel radius. For example, it is well documented that as blood flow transitions from pulsatile to non-pulsatile, so too does the scaling of vessel radii from the squared scaling (scaling exponent =2) of equation (1.3) to cubic scaling (scaling exponent =3) similar to equation (1.4) [10]. This quantitative shift would then show up as a difference between classified groups in our data that should be detectable by, and informative to, our machine learning algorithms. If the space-filling constraints and body plan of the organism primarily determine vascular architecture, then variation in scale factors for vessel lengths should best discriminate. Moreover, average properties might be shared across species while differences or variation around these average properties could reflect distinct selective pressures that can be used to discriminate types of networks and branching principles. Alternatively, some selective pressures could change the average properties yet share the same values of variation and asymmetry. Finally, if resilience to gravitational buckling determines branching form then the slenderness exponents should differentiate between those organisms susceptible to buckling (plants) and those that are not (mammals).

**Figure 2.**
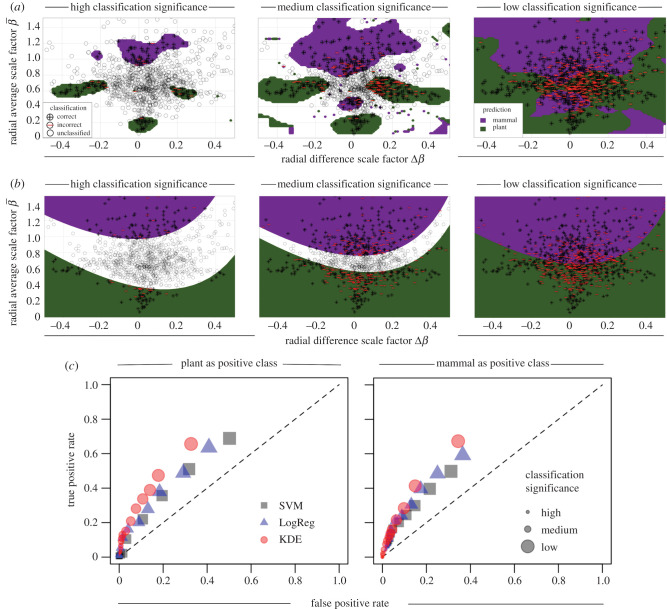
Comparison of machine learning methods. Results for the (*a*) kernel density estimation (KDE) and (*b*) logistic regression (LR) methods of classification of mammalian and plant networks. Here, both methods use the radius average and difference scale factors ($\bar{\beta }=(r_{c1}+r_{c2})/2r$, Δ*β* = (*r*_*c*1_ − *r*_*c*2_)/2*r*_*p*_). For each method, data are randomly split into training (75%) and testing (25%) groups. Following testing, classified points are binned based on predicted probability significance (or score), and comparison is made while varying the level of classification significance from high (left graphs) to low (right graphs). This procedure was reproduced 100 times, with training and testing division performed at random. (*c*) Receiver operator characteristic (ROC) curves comparing true positive rates (TPR) versus false positive rates (FPR) of classification for methods of support vector machine (SVM), LogReg and KDE for each level of classification significance. TPR and FPR are calculated in a one-versus-all framework, where TPR = true positives/(true positives + false negatives) and FPR = false positives/(false positives + true negatives). At any given level of significance, three classes exist: mammal, plant and unclassified. Thus, the one-versus-all approach means TPR and FPR are calculated separately for either ‘mammals and not mammals’ (left graph) or ‘plants and not plants’ (right graph). In both graphs, the KDE method is shown to outperform the LogReg and SVM.

**Figure 3.**
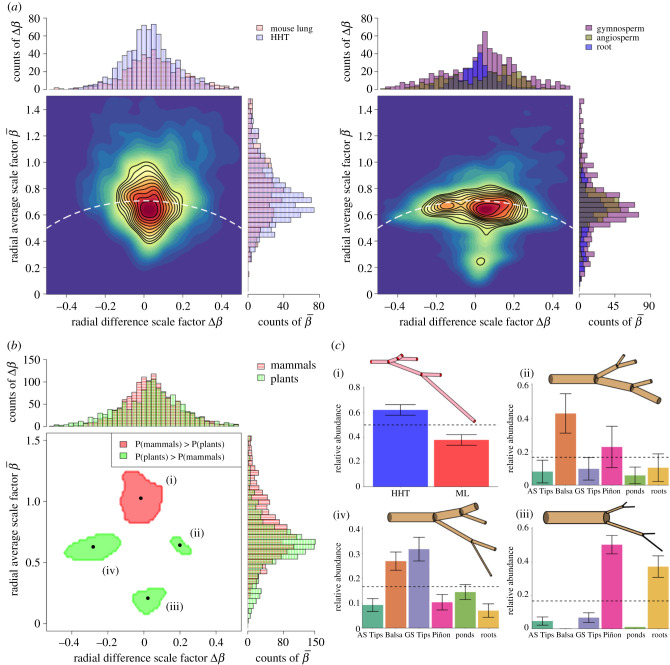
Classification based on features (the radial scale factors $\bar{\beta }$ and Δ*β*) that are related to fluid transport—blood or sap—via volume-flow rate and hydraulic resistance through networks and vessels. (*a*) Joint and marginalized distributions for the mammal (left) and plant (right) radius scale factors using the KDE method. Mammals are divided into mouse lung and HHT, and plants are divided into the groups of gymnosperms (GS), angiosperms (AS) and roots. Black contours represent lines of constant probability density, ranging from 0.5 to 0.05 in steps of 0.05. White dashed lines are graphs of the radius conservation equation for area-preservation. (*b*) Regions of significantly (*p* < 0.05) greater joint probability density for the mammals (red) or plants (green). (*c*) Representative diagrams of tree networks and bar plots of relative abundances of each group/species are presented for each region of significant classification in (*b*) (clockwise). Scale factor values for tree networks are determined by geometrically averaging over all classified data points within each significance region. Means and standard deviations for bar plots are determined by bootstrapping the KDE method 1000 times. Horizontal black dashed lines represent null expectations of relative abundances. These significance-region abundances are corroborated with global-level testing of all pairs of branching networks (see electronic supplementary material, table S1). The global-level test is a method that effectively integrates over the entire feature space to produce one singular *p*-value for the comparison [33].

**Figure 4.**
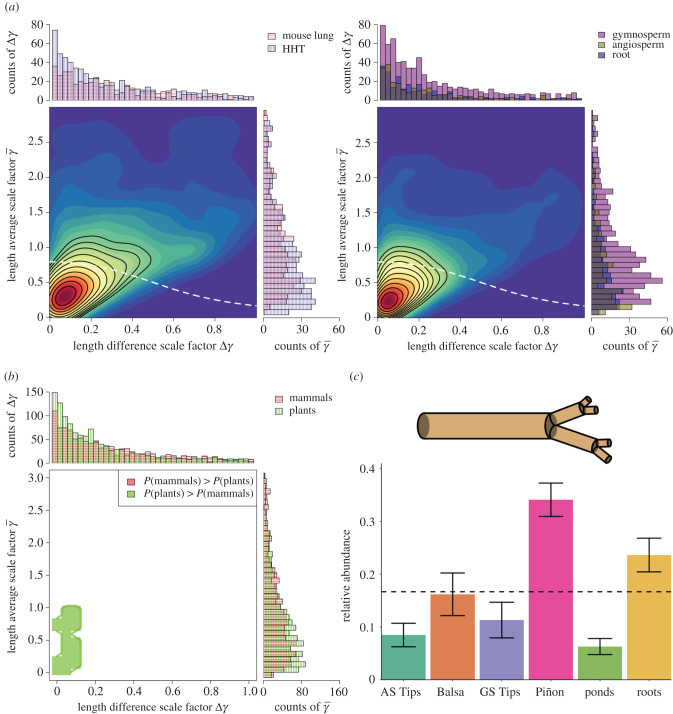
Classification based on features (the length-scale factors $\bar{\gamma }$ and Δ*γ*) that are related to costs of materials and construction for these networks as well as the extent to which they fill the space of the organisms that they are supplying with nutrients and resources. (*a*–*c*) See caption for figure 3 for description of subfigures. The significance-region abundances in (*c*) are corroborated with global-level testing of all pairs of branching networks (see electronic supplementary material, table S3).

**Figure 5.**
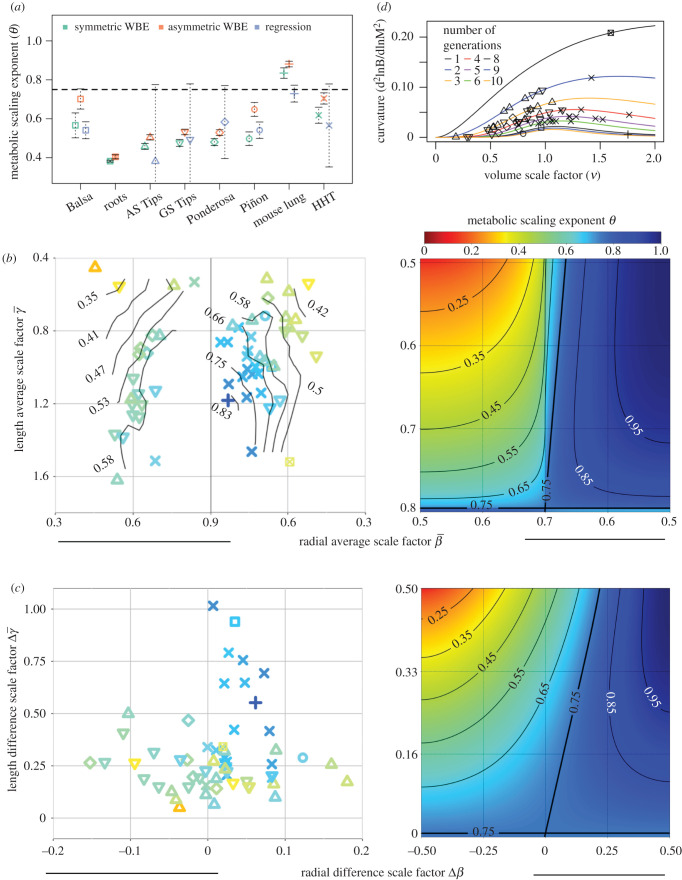
Variation in metabolic scaling exponents related to variation in branching geometry. (*a*) Comparison of *symmetric* (red) and *asymmetric* (green) estimates of metabolic scaling exponents to regression (blue) based estimates. For groups with multiple species and/or multiple individuals (AS Tips, GS Tips, Ponderosa and HHT), metabolic scaling exponents were calculated at the species/individual level when averaged. Error bars represent 95% confidence intervals. The horizontal dashed line represents a metabolic scaling exponent value of 3/4. Note that (*a*) also serves as a legend for the symbols in all other subfigures. (*b*) Empirically based estimates of metabolic scaling exponents are presented as functions of the geometrically averaged length and radial average scale factor values (left), and compared to theoretical predictions (right) reproduced from Brummer *et al.* [28]. (*c*) Analogous results are presented as in (*b*) but instead for the length and radius difference scale factors. Solid black lines represent contours of constant metabolic scaling exponent values. Axis ranges differ between empirical data and theory-based predictions due to observed deviations from conservation equations. (*d*) Curvature of metabolic rate versus mass (log–log) as a function of volume scaling ($\nu_{\mathrm{ASYM}}$) and the number of branching generations (*N*).

To test and quantify all of these possibilities, we generate distributions of our data for combinations of the raw and standardized radius and length measurements (*r*, *l*) and ${\left(r,l\right)}^{†}$ (where † represents the centred and standardized radii and lengths), the slenderness exponents (*σ*_1_, *σ*_2_), and of the symmetric and asymmetric scale factors (*β*_1_, *β*_2_, *γ*_1_, *γ*_2_, $\bar{\beta }$, $\bar{\gamma }$, Δ*β*, Δ*γ*) for the combined mammal and plant networks. We first examine the performance of several standard machine learning techniques to categorize our network data [31,32]. We use principal components analysis (PCA) to examine feature space variance (figure 1*f*,*g*), and compare the results of the nonlinear machine learning methods of support vector machine (SVM), logistic regression (LR) and kernel density estimation (KDE) (table 2 and figure 2*a*–*c*). Uncertainty is controlled for by graphing the rates of true positive detection versus false positive detection in a one-versus-all comparison between the different classifiers being used while varying the significance of classification (figure 2). See electronic supplementary material for additional detail on training and testing protocol. Upon identifying which method has the greatest overall classification success, we then examine which regions in the plant and mammal feature space drive classification and correspond to different species or tissues (figures 3 and 4). Here, we account for uncertainty by bootstrapping-with-replacement on the training and testing groups when examining better method at a fixed level of classification significance. Finally, by drawing on metabolic scaling theory—the prediction that the scaling of organism metabolism with mass is determined by vascular geometry—we examine how these different feature spaces constrain variation in estimates of the scaling exponent for organismal metabolic rate (figure 5).

**Table 2.** Global scores and effect sizes for different machine learning methods and feature spaces in classifying mammal and plant datasets.

	(*r*, *l*)	${\left(r,l\right)}^{†}$	(*σ*_1_, *σ*_2_)	(*β*, *γ*)	$\left(\bar{\beta },\bar{\gamma }\right)$	(Δ*β*, Δ*γ*)	$\left(\bar{\beta },\Delta \beta \right)$	$\left(\bar{\gamma },\Delta \gamma \right)$	$\left(\bar{\beta },\bar{\gamma },\Delta \beta ,\Delta \gamma \right)$
LogReg	0.82	0.54	0.52	0.52	0.59	0.55	0.59	0.53	0.58
SVM	0.88	0.56	0.52	0.57	0.62	0.59	0.64	0.58	0.67
KDE	2 × 10^5^****	0.049**	0	0.065	0.11	0.31	0.72***	0.13*	0.68****

The logistic regression and support vector machine scores represent the ratio of correctly classified vessels/nodes for a given feature space, and are compared to a baseline of 0.52 (as determined by the starting ratio of mammal to plant data). The kernel density estimation scores are test statistic values representing effect size in differentiating mammal from plant networks. † indicates the standardized radius and length distribution. Asterisks indicate *p*-value of KDE (no asterisk *p* > 0.01; *p ≤ 0.01; **p ≤ 0.001; ***p ≤ 0.0001; ****p = 0). All three methods demonstrate high scores (LogReg, SVM) or significant effect sizes (KDE) for the raw radial and length data due to trivial size separation (figure 1*c*) which is removed upon standardizing for zero mean and unit variance. Asymmetric scale factor feature space ($\bar{\beta },\Delta \beta$) is the relatively best-performing metric for KDE and LogReg methods, and second best for SVM. See figure 2 for absolute comparison between three methods.

## 2. Results

We demonstrate the importance of choosing theoretically informed feature spaces over raw data to classify vascular organisms relating form to function. Classification using only raw data (branch radii and lengths) results only in size-based categorization, an approach that can distinguish between a mouse lung and a Balsa tree, but is not easily applicable to similarly sized organisms or tissues (figure 1*c*). Once networks are normalized for size, distributions of the raw data are greatly overlapped [3,5] (figure 1*d*) and machine learning methods applied to the raw data cannot distinguish the networks (table 2). We thus conclude that our theoretically informed feature spaces are objectively superior at categorizing branching networks over raw data. In addition, these theoretically informed feature spaces facilitate much easier translation into known biological principles and constraints on biologic function related to blood flow, space-filling and metabolic rate.

Importantly, not all theory-motivated features improve classification. Table 2 shows that the slenderness scaling exponents (*σ*_1_, *σ*_2_) do no better than random chance. Two likely explanations are that the definitions in equation (1.5) simultaneously de-couple branching asymmetry and couple radial and length scaling. This is supported by figure 1*f*,*g*, where PCA loadings between Δ*β*, *σ*_1_ and *σ*_2_ are either directly correlated (PCA 2), or are linear combinations of each other (PCA 3 and 4). Table 2 shows that radial scaling with asymmetry ($\bar{\beta },\Delta \beta$) outperforms length scaling with asymmetry ($\bar{\gamma },\Delta \gamma$). Thus, it appears that transformations that suppress asymmetry and enhance length—such as the slenderness scaling exponents—act to obscure the defining features between the mammal and plant data considered.

Differences in machine learning performance across methods (KDE, SVM, LR) are due to the nonlinear, multivariate structure of the feature spaces being studied and the chosen machine learning method. In particular, the KDE method excels at resolving the multimodality [34] that characterizes the radial scale factors for the plant dataset (figures 2*a* and 3*a*,*b*). Since the distribution means are approximately equivalent, the SVM and LR methods are strongly influenced by outliers and the higher moments comprising the mammal dataset (figure 2*b*) [32].

Comparing across all methods and features spaces, we find that the combination of the KDE method and the average and difference scale factors for radius ($\bar{\beta },\Delta \beta$) are the most effective for classifying branching network data (figure 2 and table 1; electronic supplementary material, tables S1–S3). The fact that variation in the feature space for radial scale factors is the best has strong implications about what functional features form the major distinctions between biologic networks. *Specifically, our empirical finding of the primacy of information based on scaling ratios of radii strongly suggests that hydrodynamic principles are the primary drivers of vascular branching patterns and overall network form.*

Multiple theories of vascular networks, as well as basic physics and fluid mechanics, dictate that rates of fluid flow are largely governed by the total cross-sectional area of vessels or limbs, which can be exactly related to the ratios of scaling radii used in our feature space [7]. Importantly, theory recently developed by us demonstrates that there can exist a range of morphologies that still adhere to these area-preserving—pulsatile flow in mammals or external branching in plants—or area-increasing predictions—non-pulsatile flow in mammals [28]. By contrast, variation in the ratios of vessel lengths is more strongly tied to the ability of the vascular network to fill the body. Thus, length ratios either appear to encapsulate little important information about the differences among biologic networks, or they may not adequately capture the key properties of space filling for the architecture of vascular networks [3,5,28,35]. Having identified the best-performing feature space and machine learning method, we now delve deeper into the variation in the architecture and functional properties of vascular networks.

Focusing on the KDE method, we see that mammalian branching exhibits more area-increasing branching than plants (figure 3*b*(i)). Area-increasing branching is necessary to simultaneously increase total surface area for oxygen and metabolite transport and to slow blood flow as it travels from the heart to the capillaries and transitions from pulsatile to non-pulsatile flow, the latter phenomenon not being present in plants. However, values of $\bar{\beta }\approx 1.0$ and Δ*β* ≈ 0 represent a deviation from the theoretical predictions of Δ*β* = 0 and $\bar{\beta }=1/2^{1/3}\approx 0.794$ for the non-pulsatile flow expected in this region. This marked increase in cross-sectional area is shared by both the HHT and ML networks as indicated by the nearly null relative abundances of these two networks (figure 3*c*(i)) as well as by the insignificant *p*-value score of 0.2 from the global-level implementation of the KDE method (see electronic supplementary material, table S1). This suggests that transitions in blood flow type from pulsatile to non-pulsatile may occur across a greater range of branching generations, and begin nearer to the heart, than in current theory [7,16].

The majority of plant networks adhere to area-preservation while exhibiting a greater tendency than mammals to branch asymmetrically (specifically the Balsa, Piñon, Ponderosas and GS Tips, figure 3*a*,*b*). Within the plants we find that differentiation is driven at the species level (figure 3*c*(ii)–(iv); and electronic supplementary material, table S1), unrelated to plant categorization as angiosperm or gymnosperm. For example the Balsa, an angiosperm, is the only species present in both the positive and negative asymmetry types (figures 1*b*) as demonstrated by being the only network with its standard deviation outside the null expectation in figure 3*c*(ii),(iv). Thus, the Balsa consists of two unique branching motifs that distinguish it from GS Tips, and the Piñon and roots that have large relative abundances in one region each—the negative asymmetric branching of motif *c*(iv) and the symmetric branching of motif *c*(iii), respectively.

Mechanisms for the asymmetry and motifs observed in the plant radial scale factors are likely due to functional trait plasticity associated with light-seeking behaviour, self- and wind-induced pruning, gap-filling and other environmental stressors. However, making quantitative connections remains an open challenge [6,27,30]. For example, the slenderness scaling exponents can be calculated for all six scenarios in figure 3*c*(ii)–(iv), beginning with the first generation of child branches. Expressing equation (1.5) in terms of the average and difference scale factors:

2.1 $$\sigma_{1}=\frac{\ln(\bar{\beta }+\Delta \beta )}{\ln(\bar{\gamma }+\Delta \gamma )}\;\text{and}\;\sigma_{2}=\frac{\ln(\bar{\beta }-\Delta \beta )}{\ln(\bar{\gamma }-\Delta \gamma )}.$$

Thus, the slenderness scaling exponents for each plant motif are: *σ*_1_ = −12.3, *σ*_2_ = 1.4 for motif (ii); *σ*_1_ = −19.0, *σ*_2_ = 5.1 for motif (iii); and *σ*_1_ = −4.8, *σ*_2_ = 0.54 for motif (iv). Biomechanical theory that applies columnar (Euler beam) buckling to branching systems demonstrates that slenderness exponents of *σ* ≥ 1 are structurally advantageous for plant architectures as they push the locations of breakage points into the canopy as opposed to the trunk. Yet, the slenderness exponents we calculate for the observed motifs do not entirely agree with this framework, despite adherence of the radial scale factors in motifs (ii) and (iv) to the area-preserving branching constraint of equation (1.3). This disparity may lie in two sources: (i) the slenderness exponent formula of equation (1.5) was originally derived using symmetrically branched networks and (ii) the length scaling exponents involved in equation (1.5). The latter issue we now investigate.

Connecting length-based categorization to mechanism—the space-filling constraint of equation (1.4)—remains a challenge. The combination of the KDE method and lengthscale factor feature space ($\bar{\gamma },\Delta \gamma$) identified only one region of significance. In this region, differentiation is driven by the plants, specifically the Piñon and roots (figure 4; electronic supplementary material, table S3). This is despite the large amount of variance explained by the length-scale factors in the PCA (figure 1). The single region driving differentiation corresponds to average length-scale factors $\bar{\gamma }<1$. This effect would normally result in an increase in the slenderness exponent, equation (1.5), driving gravitationally induced buckling (self-pruning) to occur in the canopy instead of at the trunk (*σ* ≥ 1) [30]. However, median values of *σ* for both the plants and mammals were approximately 0.2, far below the needed theoretical threshold of *σ* = 1. We interpret this deviation from expected biomechanics as an indicator that the length-scale factors, as defined, are poor features for characterizing vascular or branching architecture.

The inability of the lengthscale factors to inform classification between networks suggests several scenarios. Two contrasting and extreme scenarios are that either a universal architecture or a completely random architecture is being followed by both the mammals and plants [9]. This result is unlike the radial scaling that is strongly coupled to hydraulics. Current theory suggests that the architecture associated with length scaling is guided by the principles of space-filling fractals [7,10,11,28]. However, large deviations are observed between the joint distributions of the length-scale factors and the theoretical curves determined by the space-filling conservation equation (figure 4*a*). A third scenario is that there exists a disconnect between how length-scale factors are conventionally defined in simplified models versus how they are measured in complicated natural systems. All three scenarios support the need for including missing constraints, variables, and assumptions (e.g. branching angles, multi-fractal scaling etc.), or alternative mathematical frameworks [35–39].

To better understand the physiological and biological implications of these categorizations, we examine the influence of asymmetric branching on estimates of biological rates—specifically, the metabolic scaling exponent *θ* that canonically relates metabolic rate *B* to body mass *M* as $B\propto M^{\theta }$. Previous studies spanning orders of magnitude in body mass have shown that *θ* converges on a value near 3/4, yet exhibits variation specific to mammals or plants [14–16].

To probe this variation, we use branching data to estimate metabolic scaling (figure 5) by directly accounting for network geometry and size [7,10,28],

2.2 $$\theta =\frac{\ln(2^{N})}{\ln(2^{N})+\ln(1-\nu^{N+1})-\ln(\nu^{N}(1-\nu ))},$$

where *N* is the total number of branching generations in the network and ν represents volumetric scaling—the ratio of the sum of the volumes of both child branches to the volume of the parent branch. Specification of ν allows estimation of *θ* under different model assumptions for symmetric (ν = 2*β*^2^*γ*) or asymmetric ($\nu =2{\bar{\beta }}^{2}\bar{\gamma }+4\bar{\beta }\Delta \beta \Delta \gamma +2\bar{\gamma }\Delta \beta^{2}$) branching. We also use a regression method between the number of terminal branches $N_{\mathrm{TIPS}}$ and total volume $V_{\mathrm{TOT}}$ distal to a given branch ($N_{\mathrm{TIPS}}\propto V_{\mathrm{TOT}}^{\theta }$) that does not depend directly on geometry (see electronic supplementary material). We find that asymmetric branching increases the predicted values of metabolic scaling exponents when compared to the symmetric- and regression-based methods (figure 5*a*). This is due to all networks exhibiting some length asymmetry, and more importantly suggests that previous studies have underestimated metabolic scaling exponents by not accounting for such variation [3,40,41].

To understand which different scale factors are primarily responsible for observed variation in the predicted metabolic scaling exponents we focus on the asymmetric version of equation (2.2). Estimated metabolic scaling exponents are graphed for each individual organism in terms of the average scale factors ($\bar{\beta },\bar{\gamma }$) in figure 5*b* and difference scale factors (Δ*β*, Δ*γ*) in figure 5*c*. We compare these graphs against the corresponding theoretical predictions reproduced from Brummer *et al.* [28] where we have graphed the approximate form of equation (2.2),

2.3 $$\theta \approx \frac{\ln(2)}{\ln(2)-\ln(2{\bar{\beta }}^{2}\bar{\gamma })-\ln(1+\frac{2\Delta \beta \Delta \gamma }{\bar{\beta }\bar{\gamma }}+\frac{\Delta \beta^{2}}{{\bar{\beta }}^{2}})}$$

assuming small volume scaling (*ν* < 1), generationally large networks (*N* > >1), and enforcing area-preserving and space-filling (equations (1.3) and (1.4)).

We observe a striking amount of grouping among the mammals and plants when graphing the metabolic scaling exponent *θ* versus the average radial and length-scale factors $\bar{\beta }$ and $\bar{\gamma }$ (figure 5*b*). This indicates that, of all the features and data considered, the average scale factors ($\bar{\beta }$ and $\bar{\gamma }$) are the primary determinants of variation in the metabolic scaling exponent and thus organism function.

In contrast to previous theory and importantly for understanding how diverse branching architectures could lead to universal scaling exponents, we find near constancy of the metabolic scaling exponent despite large fluctuations in length scaling (figure 5*c*). These shared exponents are likely driven by the little to no radial asymmetry observed in mammalian networks and suggests that variation in length asymmetry (Δ*γ*) in vascular networks has little influence on whole organism metabolic function in the presence of symmetric radial branching (Δ*β* = 0).

Figure 5*b*,*c* demonstrates marked deviation in the observed grouping (or lack thereof) between the empirically based predictions of metabolic scaling from equation (2.2) and the constraint-based theoretical predictions of metabolic scaling from equation (2.3). To explore this deviation, we calculate curvature between metabolic rate and mass in log–log space (electronic supplementary material). When branching networks are strictly assumed to be very large (*N* > >1) and decreasing in volume in all segments across any generation (ν < 1, equation (2.3)), we predict zero curvature, regardless of the extent of branching asymmetry. When accounting for variation in network size and volume scaling (equation (2.2)), we predict positive (concave up) curvature (figure 5*d*). These predictions are both in agreement with respiration-based studies of mammals [16], and demonstrate the need for theories of metabolic scaling that incorporate the finite size of the network. Furthermore, we predict that curvature decreases to zero with increasing network size, or generation *N*, in agreement with respiration-based studies of plants [15]. These results can be informative for future studies that simultaneously connect branching patterns and vascular data to ontogenetic- and size-based shifts in organismal metabolism. Such shifts are observed in growth and reproduction curves for tumours [42], plants [15], mammals [43] and fish [44].

## 3. Discussion

Machine learning is a powerful tool, but often considered a black box. We show that by combining machine learning with mechanistic theory it can be made more effective and provide insight into physiological mechanism. Here, we take a first step towards building that bridge by using mechanistic theory of vascular networks to choose better feature spaces. In so doing, we achieve a twofold, mutually reinforcing benefit: (i) we achieve better results for categorizing networks than if we used either the raw feature space or a mechanistically inspired feature space that predicts only one morphology (the symmetric scale factors *β* and *γ*) and (ii) results are much more interpretable. For example, the best-performing feature spaces—the asymmetric ratios of vessel and limb radii, $\bar{\beta }$ and Δ*β*—are explicitly and naturally tied to specific mechanisms—hydrodynamic constraints and resource flow—and allow for variation in form while still following these constraints. Additionally, the under-performing feature spaces—ratios of vessel and limb lengths, $\bar{\gamma }$ and Δ*γ*—identify what may be potential weaknesses in the theory and avenues for new development to provide greater specificity. Alternatively, the inability of the ratios of vessel and limb lengths to classify between mammals and plants may be pointing to broadly shared architectural principles that are not specific to mammals or plants. This is despite different mechanistic demands, such as structural support for plants.

The results of this study also serve to inform our understanding of the physiological pressures that determine convergence in organismal form and function. We find that variation in vascular based estimates of metabolic scaling exponents—in particular curvature—is primarily determined by variation in the average scale factors ($\bar{\beta }$ and $\bar{\gamma }$), symmetric radial branching and relative network size. This result helps to resolve some of the contradictory size-based observations in variation in metabolic scaling between mammals and plants [15,16]. It emphasizes the needs to develop models of vascular networks that can better account for finite-size measurements (clustered sampling versus whole network measurement), to acquire comprehensive datasets that span the entirety of the vascular branching structures being studied, and to simultaneously acquire respiration-based measurements of organismal metabolism.

In this direction, a shortcoming of our model of metabolic curvature is its complete inability to capture negative curvature. This scenario arises when examining individual growth curves in mammals, plants and tumours when the metabolic scaling exponent decreases from linear to sub-linear (typically from 1 to 3/4) [15,42,43]. Failure to capture this essential biologic feature should spur continued development of theories of metabolic scaling and vascular branching.

Finally, this work has implications for several fields, spanning bio-mechanical and physiological imaging and theory to machine learning and biomedical applications. Incorporating topological features—connectivity and loops—and branching angles could enhance categorization methods because these features provide structural integrity and redundancy to damage in plant leaves and in capillaries [45–47]. Additional measures that capture organ and organismal physiology could provide further insight and tests. Examples for mammalian tissues include flow reserve—the change in blood flow between normal and dilated vessel states—or blood perfused for a given vascular tree [48,49]. New applications of tomographic imaging and computer vision techniques to plants—light detection and ranging and positron emission tomograpahy—are greatly expanding digitized plant architecture datasets and allowing for the direct inclusion of branch angles and xylem and phloem transport measurements as part of the biological feature space [21,41,50,51]. Simultaneously, advances in medical imaging and vascular segmentation algorithms are leading to datasets of fully connected branching and blood vessel networks [52]. Such expansive datasets previously unavailable will allow for comprehensive testing of vascular branching theories where, in principle, machine learning-based motif identification could be used to digitally regenerate branching networks using iterated function systems [36,39].

Using more robust applications of machine learning methods (e.g. nonlinear dimensional reduction) and increased model complexity might help improve classification based on raw data and should improve classification using feature spaces based on theory as well. In closing, this work provides a proof-of-principle that a mechanistically based automatic classification and detection scheme for vascular networks could have application in medical diagnostics for long-term progressive disease (e.g. tumour growth). Here, classification is driven by outlier detection between vascular networks surrounding and comprising tumours compared against verified healthy vascular networks [53]. Such an application would serve as a new dimension in radiomic studies where the detection and classification of tumours based on vascular branching is wholly absent [2,54] and could provide an alternative measure of tumour growth and development [55].

## Supplementary Material

Supplementary Material: Text

## Supplementary Material

Supplementary Material: Datasets

## Supplementary Material

Supplementary Material: Python Code

## Supplementary Material

Supplementary Material: R Code

## Data accessibility

The datasets and code that support the findings of this study have been uploaded as part of the electronic supplementary material.
