# Supplementary material for: Branching principles of animal and plant networks identified by combining extensive data, machine learning and modelling
Source: J R Soc Interface. 2021 Jan 6;18(174):20200624. doi: 10.1098/rsif.2020.0624 (PMC7879751; doi:10.1098/rsif.2020.0624)
Supplement: Supplementary Material: Text [file rsif20200624supp1.pdf]

Supplementary Material

Contents

A Classifier methods **2**

B Feature space selection **3**
B.1 Raw radius and length measurements . . . 3
B.2 Scale factor feature spaces . . . 4

C Normalizing results from classifier methods **5**
C.1 Kernel density estimation . . . 5
C.2 Logistic regression and support vector machine . . . 5
C.3 ROC comparison of methods . . . 6
C.4 Data grouping . . . 7

D Derivation of exact metabolic scaling exponent formula **8**

E Derivation of curvature in metabolic rate versus mass **12**

F Kernel Density Estimation Results **13**

List of Tables

1 Table of p-values for KDE test on plant animal groups I. . . . 13
2 Table of p-values for KDE test on plant animal groups II. . . . 14
3 Table of p-values for KDE test on plant animal groups III. . . . 14
4 Table of p-values for KDE test on Ponderosa individuals. . . . 15
5 Table of p-values for KDE test on human head and torso individuals. . . . 15
6 Table of p-values for KDE test on tree tips species. . . . 16

List of Figures

1 Data classification versus sensitivity for KDE, LR, and SVM methods . . . 6
2 Sample regression analysis on mouse lung data . . . 9
3 Exact formula for metabolic scaling exponent versus volume scaling and network size . . . 10

A Classifier methods

To generate probability models using the support vector machine and logistic regression methods, approximately 75% of each combined plant and and combined mammal dataset was taken as the training sample and the remaining 25% used as the testing sample. This was done after randomization within respective datasets to minimize the chances of accidentally removing an entire individual or species. Then, using the radial scale factor feature space ($\bar{\beta}$ and $\Delta\beta$) the two groups of mammals and plants were compared using three methods: a support vector machine (SVM) model and a logistic regression (LR) model, and a non-parametric kernel density estimator (KDE) method.

The support vector machine method, a supervised machine learning algorithm for classification problems, plots data points in an n-dimensional space and draws a decision boundary by maximizing the margin between points from different classes. To generate a support vector machine model for our datasets, we used the SVC (support vector classification) function from the Python scikit-learn package. When running SVC, we used the polynomial kernel with a degree of 2.

The LR method generates probabilities of how likely certain data points are going to fall under a certain class, based on the logit function $1/(1+e^{-x})$. To produce a non-linear LR model for our two-dimensional datasets, we used the Python scikit-learn logistic regression function and added three nonlinearity columns, x_1^2, x_2^2, and $x_1 \times x_2$. All pairwise combinations were run on this LR model and the probabilities were recorded.

For both the SVM method and the LR method, a polynomial kernel was used. Through a series of tests between different kernels, including linear and radial basis function, the polynomial kernel yielded the highest accuracy score when classifying the testing data. The LR and SVM methods differ in their nonlinearities, where the LR method uses an added nonlinearity term and the SVM method uses a radial basis function kernel. The probabilities for the LR method are assigned using the logit function, and the probabilities assigned using the SVM method are based on the point distances from the decision boundary. For both of these methods, testing points are classified based on the value of their score. When using training data that is equally split between the two categories then points that receive scores of 0.5 or greater are classified as one group, and scores below 0.5 are classified as the other group.

The kernel density estimator (KDE) technique introduced by Duong et al. (*1, 2*) generates non-parametric, multi-dimensional probability distributions, $\mathbb{P}_i(\mathbf{x})$, of vascular traits, **x**, for each testing group, $i = A, B$. These distributions are then compared against one another for their extent of uniqueness, or non-overlap, represented by the test statistic $T = \int [\mathbb{P}_A(\mathbf{x}) - \mathbb{P}_B(\mathbf{x})]^2 d\mathbf{x}$. This test can be thought of as a multi-dimensional generalization of the two-sample Kolmogorov-Smirnov (KS) test, where significance of classification is conventionally communicated through p-values. We use the nominal threshold of $p \leq 0.05$ as a threshold for significance when using the KDE method.

The KDE method can be applied both globally (*1*) and locally (*2*). At the global level, the test statistic T is calculated as described above (and in more detail in (*1*)), and converted to a p-value using standard tables. While $p = 0.05$ is used as the nominal threshold for significance, it should be noted that p values ranging orders of magnitude in size from $p = 10 \times 10^{-2}$ to $p = 10 \times 10^{-20}$ are observed, and thus interpreted as relative levels of significance. The local application of the KDE method is effectively an inversion of the calculation for the test statistic T. Here, one sets the desired threshold for significance, or the value of the test statistic T, then calculates which regions in the vascular trait-space, **x** correspond to the chosen test statistic.

B Feature space selection

Here we describe our methods for selecting the feature space. We tested each machine learning method on a variety of vascular trait feature spaces. Specifically, we tested the feature spaces of: raw and standardized diameter and length measurements; the slenderness scaling exponents, σ_1, and σ_2; the symmetric WBE diameter and length scale factors, β and γ; and five combinations of the asymmetric scale factors. The five combinations of asymmetric scale factors were: the average scale factors $\bar{\beta}$ and $\bar{\gamma}$; the difference scale factors $\Delta\beta$ and $\Delta\gamma$; the radial scale factors $\bar{\beta}$ and $\Delta\beta$; the length scale factors $\bar{\gamma}$ and $\Delta\gamma$; and all four asymmetric scale factors $\bar{\beta}, \bar{\gamma}, \Delta\beta$, and $\Delta\gamma$. These results are presented in Table **??**. We also conducted a principle components analysis (PCA) on the asymmetric scale factors to identify which combinations of scale factors explain the most variance in the datasets (Figure **??f-g**). Performing a PCA on all vascular variables is non-trivial due to the non-random presence of empty-cells in the dataset (*3*).

B.1 Raw radius and length measurements

Prior to transforming the radial and length measurements of our vascular datasets as motivated by scaling theory (*4–6*), we applied all three of the logistic regression (LR), support vector machine (SVM), and kernel density estimator (KDE) methods on the raw, untransformed data. This was done in two ways, first on the data after all measurements were converted to *meters* (Figure **??c**), then again after performing a standardized transformation by translating each species- or group-level distribution to be centered about zero, and then normalizing by the respective standard deviations (Figure **??d**).

Classifying vascular data based on metric size is both trivial and uninformative. Small networks (mouse lung) are clearly distinct from large networks (whole trees), and varying degrees of overlap will exist in the intermediate range of all other networks (Figure **??c**). All three methods yield significant global classification scores, as demonstrated by the example scores of LR: 82%, SVM: 88%, and KDE: T-stat $\approx 2 \times 10^5$ found in Table **??**. One can certainly argue that classifying networks in this manner is possible, even though it is simply demonstrating that networks of differing size are distinguishable. However, this approach does not provide an obvious path toward understanding at a mechanistic level why certain patterns are observed beyond the simple size-based classification. Furthermore, when applying these methods for the purposes of disease detection one is oftentimes examining healthy and diseased tissues that are of similar size classes. Thus, the utility of size-based classification is rendered moot, and we must transform.

The most common transformation is to standardize the data such that the distribution means are centered about the origin and to normalize by the variance (*7, 8*) (Figure **??d**). While this approach has the benefit of removing the global size-based hierarchy between the networks, it fails to address the common pattern of local size-based hierarchy that is commonly found within networks (*4, 5, 9*). Specifically, the abundance-size distribution of vessels in a vascular network is approximately exponential due to the fact that the vessels, on average, decrease in size at every bifurcation. Thus, classification becomes immediately obscured, as demonstrated by the noticeably decreased example scores of LR: 54%, SVM: 56%, and KDE: T-stat $= 0.049$ (with a corresponding p-value less than 0.001) in Table **??**. The fact that the KDE method still yielded a significant score is a consequence of this method excelling at detecting multimodality between distributions. Yet, even though the KDE method does yield a significant score, the effect size is negligible and we still have the original problem of how to interpret the results, only now it is further compounded after having performed the standardization transformation. It is possible to consider alternative transformations based on mechanical principles—flow-rates and pressures (*10*)—yet the problem of the hierarchy of sizes returns, only now with different physical units. As a consequence of these complications in analyzing raw data, we turn to classification based on scale factor variables as guided by the metabolic scaling theory literature.

B.2 Scale factor feature spaces

To investigate the scale factor feature spaces, all three machine learning methods were tested on different combinations of candidate feature spaces, and a principle component analysis (PCA) was conducted on the asymmetric scale factor feature space. Results from the application of different machine learning methods are presented in Table **??** and graphs from the PCA are presented in Figure **??f-g**. Table **??** shows that the top two combinations of features for classifying animal and plant vascular networks are the set of all asymmetric scale factors ($\bar{\beta}, \Delta\beta, \bar{\gamma}, \Delta\gamma$) and the radial scale factors ($\bar{\beta}, \Delta\beta$) . That the set of all asymmetric scale factors performs the best is not surprising as this is the most comprehensive set of data for the vascular networks considered. However, it does not help to identify which subset of variables are primarily responsible for classification over other variables. To do that, we focus our attention to the PCA results.

A common feature of vascular network data is the large variation observed in the the scaling of branch lengths (*4, 5, 9*), often times exhibited over many orders of magnitude. In Figure **??f** we see that the first principle component explains 34.2% of the variance in the data, and is composed primarily of the length scale factors ($\bar{\gamma}, \Delta\gamma$). Interestingly, the length scale factors are not powerful for classification purposes (see Table **??** and Figure 4 in the main text), even though they are responsible for much of the variance in the data. On the other hand, we find that correlations between the radial scale factors account for 17.3% and 16.4% of the variance through the second and third principle components. This result, in combination with the fact that the radial scale factors had the best global classification scores in Table **??**, led to the selection of the radial average and difference scale factors as the final choice for the feature space.

C Normalizing results from classifier methods

While the three machine learning methods tested effectively perform the same task (e.g. classification of data), the manner in which this is done varies significantly, and thus requires careful consideration when trying to compare results. This difficulty is compounded by the inclusion of a variable classification sensitivity level. Here we describe the process used to standardize classification output from the three methods of Kernel Density Estimation (KDE), Support Vector Machine (SVM), and Logistic Regression (LR). Only one variable feature space was used for the comparison between methods, and that was the radial scale factor feature space ($\bar{\beta}, \Delta\beta$). This feature space was chosen as it performed best for classification across all methods (Table **??**, as well as explaining up to 33% of the variance in all of the asymmetric scale factor variables (Figure **??f-g**). The testing groups used were that of mammal and plant. These were chosen to increase the performance of methods reliant on dataset size for training and testing. Finally, we present receiver operating characteristic (ROC) curves that are used to perform the final comparison once standardization has been achieved.

C.1 Kernel density estimation

The non-parametric kernel density estimation procedure put forth by Duong et al. (*1, 2*) tests for uniqueness and overlap between two different probability distributions generated from empirical data. Probability distributions, $\mathbb{P}_i(\mathbf{x}, \mathbf{X})$ are defined on a discretized feature space, where **x** represents the discretized coordinates in the feature space, **X** represents actual empirically measured values, and $i = A$ or B represents our known classifier. When performing the local significant difference test, one must first set the p-value that denotes "significance". Conventionally this value is set at either $p = 0.01$ (as done for the analysis presented in the main text) or $p = 0.05$. The KDE method then identifies regions of the feature space where the squared-difference between the probabilities, $\mathbb{P}_i(\mathbf{x}, \mathbf{X})$, is less than the selected significance level. Finally, by examining which probability, $\mathbb{P}_i(\mathbf{x}, \mathbf{X})$, is greatest within each region, one can identify which classifier is driving differentiation. Thus, one can measure the performance of the KDE method at a given significance level by counting the number of correctly and incorrectly classified points with respect to each region (Figure S1).

By varying the p-value one can vary the relative size of the classified regions of the test to examine the efficacy of classification (Figure S1). For the KDE method, the p-value was varied by integer orders of magnitude from 10^{-12} to 10^{4} to ensure that the limiting scenarios of zero points classified and all points classified were contained. Then, for each sensitivity level, correctly and incorrectly classified points were tallied for further analysis and comparison in a one-versus-all framework.

C.2 Logistic regression and support vector machine

The logistic regression (LR) and support vector machine (SVM) methods represent two approaches at using supervised machine learning methods for classification (*7, 11*). These methods differ from the KDE approach by using a training set of data to partition the feature space into two separable regions (separated by the decision boundary). Then, points from a testing set of data are assigned a classification score based on their positions in the feature space with respect to the decision boundary. In Figure S1 are graphs of results from the LR method and the SVM method. In these examples, the decision boundary corresponds to a probability score of 0.5, and any testing point with a score between 0 and 0.5 is classified as a plant, while a score between 0.5 and 1 is classified as a mammal.

To compare to the output of the KDE approach, an analogue of significance to the KDE approach must be defined for the LR and SVM approaches. In the context of the LR and SVM approaches this was done by defining significance as the distance a probability score is from 0.5. Regions of equal significance were determined by binning along the probability score axis. Thus, points with probability scores in the ranges of $[0, 0.05]$ for mammals or $[0.95, 1]$ for plants

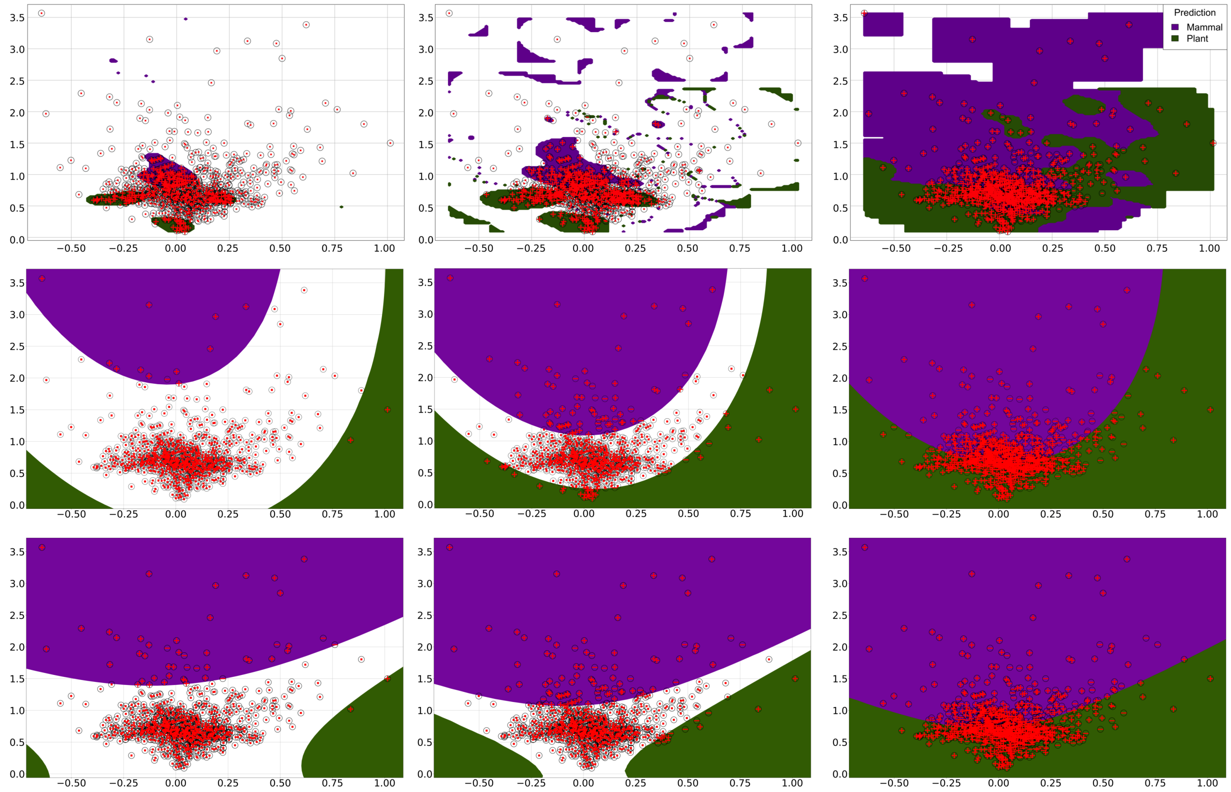

Figure S1: Data classification versus significance thresholds for KDE (top row), LR (middle row), and SVM (bottom row) methods. Significance thresholds vary from high (left column), to medium (middle column), to low (right column). Horizontal axes all correspond to the radial difference scale factor ($\Delta\beta$), while vertical axes all correspond to the radial average scale factor ($\bar{\beta}$).

would all be characterized as equally, highly significant predictions (or in terms of the KDE method, would correspond to the next lowest possible p-value). The bins were then successively enlarged to reflect a decrease in test significance. So, for mammals, the bins used were $[0, 0.05]$, $[0, 0.1]$, $[0, 0.15]$, $[0, 0.2]$, $[0, 0.25]$, $[0, 0.3]$, $[0, 0.35]$, $[0, 0.4]$, $[0, 0.45]$, $[0, 0.5]$. For plants, the bins used were $[0.95, 1]$, $[0.9, 1]$, $[0.85, 1]$, $[0.8, 1]$, $[0.75, 1]$, $[0.7, 1]$, $[0.65, 1]$, $[0.6, 1]$, $[0.55, 1]$, $[0.5, 1]$. In Figure S1 are graphs of results from the LR and SVM methods showing the successive binning approach. Finally, for each binned region corresponding to varying levels of significance, correctly and incorrectly classified points were identified for comparison in a one-versus-all framework.

C.3 ROC comparison of methods

To finally compare the three methods of kernel density estimation (KDE), logistic regression (LR), and support vector machine (SVM), we graphed receiver operating characteristic (ROC) curves of the results of the methods as sensitivities were varied (*7, 11*). ROC curves are graphs of a methods true positive rate (TPR) versus its false positive rate

(FPR). Due to the manner in which we are comparing machine learning methods, we technically have three classes for any given significance level: mammals, plants, and undetected. Thus, we must use a one-versus-all framework for calculating true and false positives. In calculating the TPR and FPR, we first choose which category we are "detecting", say plants, then the TPR and FPR can be calculated as,

$$TPR = \frac{N_{plant+}}{N_{plant+} + N_{not\ plant-}}$$
$$FPR = \frac{N_{plant-}}{N_{plant-} + N_{not\ plant+}} \tag{1}$$

where $N_{plant\pm}$ corresponds to the number of data points correctly (or incorrectly) classified as plant as denoted by the $+$ sign ($-$ sign) within the green plant contours in Figure **??**, and $N_{not\ plant\pm}$ corresponds to the number of data points correctly (or incorrectly) classified as not plant external to the green plant contours. A TPR and an FPR is then calculated for each level of significance (defined by a p-value for the KDE approach, or a probability bin for the LR or SVM approaches). Finally, the ROC curve can be graphed, as presented in Figure **??c**.

Conventionally, when comparing classification schemes using an ROC curve, the best method is identified as whichever method sits most in the upper-left corner of the graph, or which ever has the greatest "area under the curve" (AUC), as this represents a maximal true positive rate and a minimal false positive rate. To use the ROC curves for the different methods of KDE, LR, and SVM, we can use Eq. (1) and its mammal classifying analogue, resulting in Figure **??c**. Here we can observe that overall the KDE method outperforms the LR and SVM methods as having either a lower FPR for a given TPR (plant as positive class graph on left in Figure **??c**) or a greater TPR for a given FPR (mammal as positive class graph on right in Figure **??c**).

C.4 Data grouping

Once selection of the classifier was made, the subgroups of datasets were prepared for higher resolution classification. Using the KDE method, individual HHT and ponderosas were found indistinguishable, as were species within the GS Tips and AS Tips groups, hence their final merging into larger datasets (see Tables S4-6). We obtained 8 different major species/groups: HHT, mouse lung, Balsa, Piñon, Ponderosa, GS Tips, AS Tips, and Roots, each with 6 recorded variables: $\bar{\beta}, \bar{\gamma}, \Delta\beta, \Delta\gamma$, and the merging of β_1 and β_2 into a single distribution as well as for γ_1 and γ_2. Specific groupings of the scale factor variables used were: $(\beta_1, \beta_2; \gamma_1, \gamma_2)$ as a two-dimensional distribution representative of the *symmetric* formalism; $(\bar{\beta}, \bar{\gamma}, \Delta\beta, \Delta\gamma)$ as a four-dimensional distribution representative of the *asymmetric* formalism; $(\bar{\beta}, \bar{\gamma})$ as a two-dimensional distribution for *average* scaling; $(\Delta\beta, \Delta\gamma)$ as a two-dimensional distribution for *difference* scaling; $(\bar{\beta}, \Delta\beta)$ as a two-dimensional distribution for radial scaling; and $(\bar{\gamma}, \Delta\gamma)$ as a two-dimensional distribution for length scaling. For the full list of comparison results using the KDE method, see Tables S1-3.

D Derivation of exact metabolic scaling exponent formula

Here we present a derivation of the metabolic scaling exponent under the general assumptions of the West, Brown, Enquist model for vascular branching (*6, 12, 13*). This approach deviates from conventional derivations in that zero approximations will be made regarding network size or the particulars of volume scaling outside of the bounds of self-similarity. Some discussion will be presented at the end regarding how network geometry can be included in terms of symmetric or asymmetric branching.

The typical starting point for modeling metabolic scaling is Kleiber's Law (*14*), the empirically motivated, power-law relationship between organismal basal metabolic rate and mass, expressed as,

$$B = B_0 \left(\frac{M}{M_0}\right)^{\theta} \tag{2}$$

Here B represents the measured metabolic rate, M the mass, θ the metabolic scaling exponent observed to cluster around 3/4 (*14, 15*), and B_0 and M_0 normalization constants. Treating metabolic rate as the combined sum of metabolism of every terminal branch in an organism, we can substitute $B = B_{\text{TIP}} N_{\text{TIPS}}$, where B_{TIP} represents total metabolism per terminal branch and N_{TIPS} the total number of metabolizing terminal branches. Doing so results in,

$$B_{\text{TIP}} N_{\text{TIPS}} = B_0 \left(\frac{M}{M_0}\right)^{\theta} \tag{3}$$

Next, mass is substituted with total volume. The validity of this substitution is the result of having optimized the geometrical scaling of a hierarchically branching vascular network against the dual demands of hydrodynamic resistance to fluid flow and fractal space-filling (*6, 12, 13*). Performing the substitution gives us,

$$N_{\text{TIPS}} = \left(\frac{V_{\text{TOT}}}{V_0}\right)^{\theta} \tag{4}$$

where we have cancelled out B_{TIP} with B_0. At this point it is important to pause and recognize Eq. 4 as a method by which one can estimate the metabolic scaling exponent in a vascular organism free from explicitly imposing assumptions regarding network geometry via symmetric or asymmetric branching. One powerful aspect of Eq. 4 is that it can be applied recursively on any individual vascular branching network. Specifically, for every branch (or vessel) in a network, the total number of distal terminal branches can be represented by N_{TIPS}, and the total volume of all distal branches are represented by V_{TOT}. Thus, a standard major axis regression analysis can be performed to determine the value of θ that corresponds to an individual organism as per this model. An example of such an analysis being performed on the mouse lung data set is presented in Figure S2.

It is at this point that we must specify in greater detail the functional form of the total volume of the vascular network, V_{TOT}. Assuming that we are working with a strictly self-similar, hierarchically branching, pipe-like model, then the total volume of the network can be expressed as

$$V_{\text{TOT}} = V_{\text{N,TOT}} \sum_{j=0}^{N} \nu^{-j} \tag{5}$$

where j and N represent the jth and N^{th} generations of the network, $V_{\text{N,TOT}}$ is the total volume of the terminal (N^{th}) generation, and ν represents the ratio of total volume from sibling branches to their respective parent branch. For example, in a bifurcating symmetric network, $\nu = 2(\pi r_{j+1}^2 l_{j+1})/(\pi r_j^2 l_j) = 2\beta^2\gamma$. Recognizing Eq. 5 as a geometric series, we can write it's exact form as,

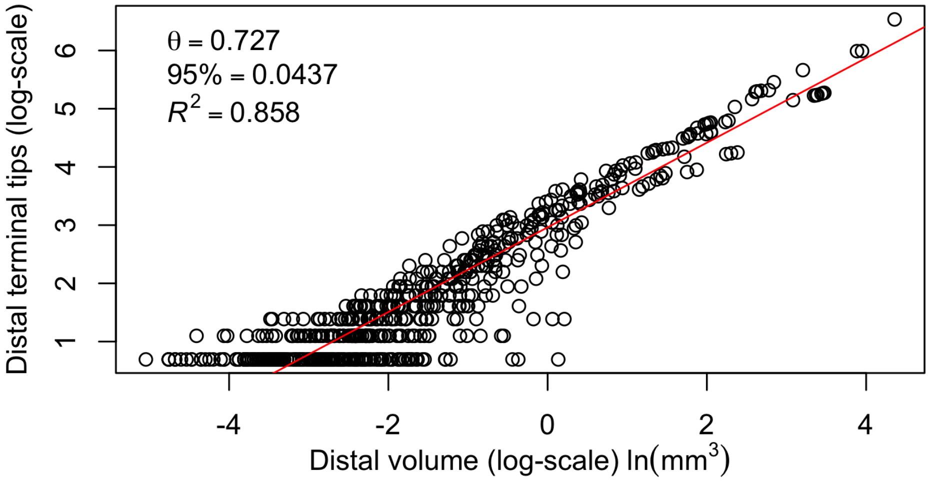

Figure S2: Sample regression analysis of distal terminal tips to distal vessel volume on mouse lung data using Standard Major Axis (SMA) regression. The fit line corresponding to the reported value of θ is represented in red.

$$V_{\text{TOT}} = V_{\text{N,TOT}} \frac{1 - \nu^{-(N+1)}}{1 - \nu^{-1}} \tag{6}$$

which is valid for all values of ν except for $\nu \approx 1$. It is not uncommon to find individual organisms with $\nu \approx 1$. This scenario is problematic if using the above formula due to its asymptotic nature. However, this problem can be remedied by using L'Hôpital's rule, producing the piecewise result,

$$V_{\text{TOT}} = \begin{cases} V_{\text{N,TOT}} \dfrac{1 - \nu^{-(N+1)}}{1 - \nu^{-1}} & \text{for } \nu \neq 1 \\ V_{\text{N,TOT}} \dfrac{N+1}{\nu^N} & \text{for } \nu \approx 1 \end{cases} \tag{7}$$

Upon substituting Eq. 7 into Eq. 4, we arrive at the following exact, piecewise function for the metabolic scaling exponent,

$$\theta = \begin{cases} \dfrac{\ln(2^N)}{\ln(2^N) + \ln(1 - \nu^{N+1}) - \ln(\nu^N(1-\nu))} & \text{for } \nu \neq 1 \\ \dfrac{\ln(2^N)}{\ln((N+1)2^N) - N\ln(\nu)} & \text{for } \nu \approx 1 \end{cases} \tag{8}$$

where we set $V_{\text{N,TOT}} = N_{\text{TIPS}} V_{\text{TIP}}$, V_0 was cancelled out with V_{TIP}, and we have restricted ourselves to considering strictly bifurcating networks such that $N_{\text{TIPS}} = 2^N$. One benefit of Eq. 8 is that we can easily examine the functional relationship between metabolic rate and the scaling of volume in a general sense, as presented in Figure S3. Examining the influence of symmetric or asymmetric branching can then be done separately.

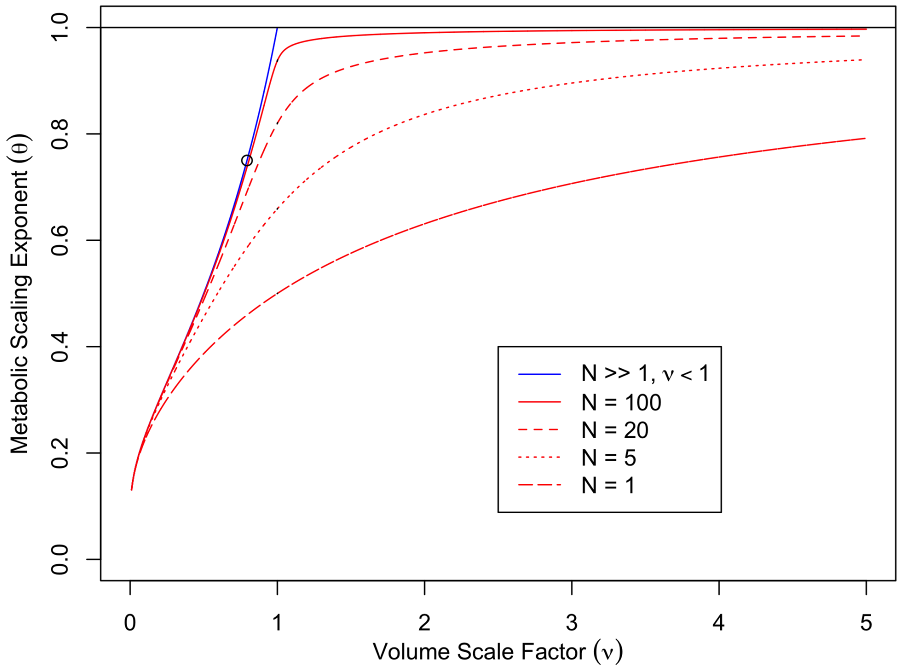

Figure S3: Exact formula for metabolic scaling exponent versus volume scaling and network size. In red are presented different exact metabolic scaling exponent curves corresponding to different values of total network generations N. In blue is the curve for the large network size (large N) and sub-linear volume scaling ($\nu < 1$) approximation. The black circle represents the particular scenario of 3/4 metabolic scaling corresponding to the symmetric WBE predicted value for $\nu = n\beta^2\gamma = 1/2^{1/3}$.

Several important features are present in Figure S3. Most notably is the effect that network size, N, has on varying the sensitivity of the metabolic scaling exponent to the value of the volume scale factor, ν. For all values of network size, the metabolic scaling exponent is bounded between zero and one. For the case of large networks ($N = 100$ in Figure S3), the metabolic scaling exponent nearly reaches the asymptotic value of $\theta = 1$ when $\nu = 1$, where as for smaller size networks (lesser values of N), the rate of increase in θ is markedly less. This result lends support to the argument that the approximate form of Eq. 8, namely $\theta \approx \ln(2)/(\ln(2) - \ln(2\beta^2\gamma))$ for a symmetrically branching network, holds in the limit that $N >> 1$ and $\nu < 1$. This approximate form of θ is presented in Figure S3 by the solid, blue line.

A second important result present in Figure S3 is the fact that all network sizes can take on the empirically observed value of $\theta = 3/4$ as long as ν is large enough. This result supports previous arguments regarding transitions in blood flow related to shifts in the scaling of radii. In these circumstances, the radial scaling transitions from cross-sectional area-preserving ($\beta^2 = 1/2$ for a symmetrically bifurcating network) to area-increasing ($\beta^3 = 1/2$). Due to the conservation fluid flow-rates across branching junctions, this latter scaling acts to slow the flow of blood. However, a simultaneous result of changing the scaling of radii in such a way is to increase the volume scale factor, assuming that there is no change in the scaling of lengths. Thus, this predicted trade-off between decreasing network size and increasing volume scaling would appear consistent with the biological demands associated with the cardiovascular

system in mammals. As for plants, ontogenetic shifts in metabolic scaling (as measured through total respiration) have been reported between sapling and mature trees and shrubs, yet not in the context of the measurement of vascular branching traits (*16*). Thus, it would be interesting to see if future studies corroborate trade-offs between metabolic scaling exponents, vascular branching traits, and network size.

In using Eq. 8, one must specify (and thereby either measure or estimate) the total number of branching generations in a vascular network. Unfortunately this is not a particularly straightforward task. With the conventional WBE generational labeling scheme, the generation index is advanced once a branching occurs for all branches within the previous generation. For highly asymmetric networks this can result in having branches of wildly differing size be members of the same generation. Alternative labelling schemes have been proposed outside the context of the WBE framework, specifically that of Horton-Strahler (*17, 18*). While capable of handling moderate levels of asymmetry, one can show that the Horton-Strahler labelling scheme is insufficient at handling networks resembling the fishbone branching pattern. For the purposes of this work, we adopt an approach based on the asymptotically symmetric expectation that for a network with N generations, there should be approximately 2^N terminal branches. Thus, from a count of the number of terminal branches, N_{TIPS}, one can estimate the number of generations as $N = \ln(N_{\text{TIPS}})/\ln(2)$.

Lastly, in the main texts standard errors are reported for calculated values of the metabolic scaling exponent in Figure 4**a**. These errors were determined from standard methods, namely $\sigma_\theta^2 \approx \left\|\frac{\partial\theta}{\partial\nu}\right\|^2 \sigma_\nu$, where σ_ν is determined from the chosen symmetric or asymmetric scale factor variables.

E Derivation of curvature in metabolic rate versus mass

Here we present a derivation of the curvature in the metabolic rate, B, of an organism in terms versus its mass, in log-log space. Beginning again with Kleiber's Law after having linearized the equation, we have,

$$\ln(B) = \ln(B_0) + \theta \ln\left(\frac{M}{M_0}\right) \tag{9}$$

As we are interested in examining the mass related curvature in metabolic rate, we must evaluate $\partial^2 \ln(B)/\partial(\ln(M))^2$. Since θ is effectively a mass-dependent quantity through its dependence on network size via total generation number N, we need to find an appropriate substitute for mass. Assuming that the total mass of a vascular organism can be expressed asymptotically as the sum total of all cells serviced by the vascular architecture, then $M \approx M_{\text{TIP}} 2^N$. Thus, we can build a derivative operator as,

$$\frac{\partial}{\partial \ln(M)} = \frac{1}{\ln(2)} \frac{\partial}{\partial N} \tag{10}$$

Using this operator, curvature in metabolic rate can be expressed as follows,

$$\frac{\partial^2 \ln(B)}{\partial(\ln(M))^2} = \frac{2}{\ln(2)} \frac{\partial \theta}{\partial N} + \frac{N}{\ln(2)} \frac{\partial^2 \theta}{\partial N^2} \tag{11}$$

where we have maintained the explicit notation for mass, M, on the left hand side as a reminder of what the quantity on the right represents. Now the problem is reduced to calculating derivatives of the metabolic scaling exponent θ with respect to total number of generations N. An important and immediate consequence of Eq. 11 is that the approximate ($\nu < 1$) and asymptotic ($N >> 1$) version of Eq. (8), $\theta \approx \ln(2)/\left[\ln(2) - \ln(\nu)\right]$, results in zero curvature due to its invariance in relative network size. This prediction is consistent with alternative parameterizations of curvature in metabolic scaling (*19*), and could inform observed deviations from previous theoretical predictions of metabolic scaling exponents as discussed in the main text.

Focusing on the version of Eq. (8) for $\nu \neq 1$, we find that the curvature in B can be expressed finally in the relatively compact form of,

$$\frac{\partial^2 \ln(B)}{\partial(\ln(M))^2} = \frac{1}{x^4}\left\{2x^3 + 2N^2 x \left(\frac{\partial x}{\partial N}\right)^2 - 4Nx^2 \frac{\partial x}{\partial N} - N^2 x^2 \frac{\partial^2 x}{\partial N^2}\right\} \tag{12}$$

where $x = \ln\left(V_{\text{TOT}}/V_{\text{TIP}}\right)$. Graphs of Eq. 12 are presented in the main text, where we find that curvature is predicted to always be positive, consistent with previous studies based on mammalian respiration (*19*), but inconsistent with studies based on plant respiration (*16*). Noting the form of x, we can identify that $x \propto N$, thus curvature is proportional to $1/N$ and decreases with an increasing number of generations. This network size-based suppression of curvature has been reported in relation to plant respiration (*16*).

These results demonstrate a current knowledge gap within the field of metabolic scaling, and motivate the simultaneously conducting respiration-based measurements of metabolic rates and vascular imaging to better synchronize prediction with observation.

Table S1 | Results of pairwise classification of plants and animal groups using asymmetric scale factors related to hydraulics and fluid transport ($\bar{\beta}, \Delta\beta$) or the strictly symmetric branching scale factors (β, γ)

ASYM / SYM	HHT*	Mouse Lung	Balsa	Piñon	Ponderosa*	GS Tips*	AS Tips*	Roots
HHT*	–	2×10^{-1}	6×10^{-13}	1×10^{-4}	3×10^{-2}	9×10^{-9}	1×10^{-1}	2×10^{-2}
Mouse Lung	6×10^{-2}	–	5×10^{-18}	7×10^{-3}	7×10^{-5}	2×10^{-19}	2×10^{-2}	2×10^{-6}
Balsa	1×10^{-1}	4×10^{-4}	–	1×10^{-16}	6×10^{-2}	1×10^{-6}	6×10^{-2}	6×10^{-13}
Piñon	3×10^{-7}	3×10^{-6}	2×10^{-7}	–	4×10^{-13}	3×10^{-56}	2×10^{-5}	2×10^{-13}
Ponderosa*	2×10^{-1}	7×10^{-2}	2×10^{-3}	4×10^{-4}	–	9×10^{-5}	3×10^{-1}	1×10^{-4}
GS Tips*	9×10^{-3}	3×10^{-2}	8×10^{-4}	1×10^{-3}	1×10^{-1}	–	7×10^{-12}	2×10^{-11}
AS Tips*	2×10^{-1}	1×10^{-1}	7×10^{-3}	2×10^{-2}	4×10^{-1}	3×10^{-2}	–	5×10^{-3}
Roots	1×10^{-1}	6×10^{-3}	2×10^{-3}	2×10^{-5}	1×10^{-1}	3×10^{-2}	3×10^{-1}	–

Table of *p*-values for pairwise KDE testing on plant and animal groups comparing different feature spaces. The lower diagonal corresponds to scale factors associated with strictly symmetric branching (β, γ), while the upper diagonal corresponds to scale factors associated with hydraulics and asymmetric radial branching $(\bar{\beta}, \Delta\beta)$. Significant classification is defined as occurring when $p < 0.05$. Datasets with multiple individuals that were aggregated are indicated with asterisks (*). Of note is the universally enhanced ability of the radial scale factor feature space to differentiate between groups when compared to the symmetric scale factor feature space.

F Kernel Density Estimation Results

In this section we present the full results from the kernel density estimation procedure spanning all combinations classification features related to geometric scaling variables.

Table S2 | Results of pairwise classification of plant and animal groups using asymmetric scale factors that are related to space-filling only ($\bar{\gamma}, \Delta\gamma$) or space-filling and hydraulics ($\bar{\beta}, \Delta\beta, \bar{\gamma}, \Delta\gamma$).

LENGTH \ ALL	HHT*	Mouse Lung	Balsa	Piñon	Ponderosa*	GS Tips*	AS Tips*	Roots
HHT*	–	7×10^{-2}	8×10^{-1}	6×10^{-3}	1×10^{-1}	1×10^{-2}	3×10^{-2}	8×10^{-3}
Mouse Lung	1×10^{-1}	–	1×10^{-1}	2×10^{-3}	4×10^{-1}	4×10^{-1}	9×10^{-2}	9×10^{-6}
Balsa	9×10^{-5}	2×10^{-5}	–	2×10^{-2}	1×10^{-2}	1×10^{-3}	1×10^{-2}	3×10^{-2}
Piñon	4×10^{-11}	4×10^{-8}	1×10^{-8}	–	3×10^{-1}	6×10^{-2}	5×10^{-1}	3×10^{-1}
Ponderosa*	8×10^{-3}	9×10^{-3}	7×10^{-3}	6×10^{-5}	–	3×10^{-1}	5×10^{-1}	1×10^{-1}
GS Tips*	2×10^{-4}	6×10^{-4}	1×10^{-3}	1×10^{-5}	2×10^{-1}	–	3×10^{-1}	9×10^{-4}
AS Tips*	5×10^{-2}	2×10^{-2}	1×10^{-1}	1×10^{-2}	4×10^{-1}	1×10^{-1}	–	3×10^{-1}
Roots	3×10^{-4}	1×10^{-4}	8×10^{-8}	1×10^{-3}	2×10^{-2}	1×10^{-3}	4×10^{-2}	–

Table of *p*-values for pairwise KDE testing on plant and animal groups comparing different features. The lower diagonal corresponds to scale factors associated with asymmetric branching related to space-filling ($\bar{\gamma}, \Delta\gamma$) while the upper diagonal corresponds to scale factors associated with the combined asymmetric feature spaces related to both hydraulics and space-filling ($\bar{\beta}, \Delta\beta, \bar{\gamma}, \Delta\gamma$). Significant classification is defined as occurring when $p < 0.05$. Datasets with multiple individuals that were aggregated are indicated with asterisks (*). In this scenario we see that nearly all groups (excluding the AS Tips) are more differentiable when using all scale factors for the feature space when compared to the length scale factor feature space.

Table S3 | Results of pairwise classification of plant and animal groups using asymmetric scale factors that are related to hydraulics and space filling and that are associated with the central means of asymmetric branching ($\bar{\beta}, \bar{\gamma}$) or scale factors associated with variation in asymmetric branching ($\Delta\beta, \Delta\gamma$).

DIFF \ AVE	HHT*	Mouse Lung	Balsa	Piñon	Ponderosa*	GS Tips*	AS Tips*	Roots
HHT*	–	4×10^{-1}	4×10^{-3}	5×10^{-3}	3×10^{-1}	3×10^{-1}	2×10^{-1}	4×10^{-2}
Mouse Lung	2×10^{-1}	–	3×10^{-2}	1×10^{-3}	3×10^{-1}	3×10^{-1}	3×10^{-1}	7×10^{-3}
Balsa	3×10^{-2}	8×10^{-7}	–	2×10^{-4}	2×10^{-1}	8×10^{-2}	7×10^{-2}	1×10^{-5}
Piñon	4×10^{-2}	1×10^{-2}	3×10^{-6}	–	1×10^{-1}	8×10^{-2}	2×10^{-1}	8×10^{-2}
Ponderosa*	3×10^{-2}	1×10^{-3}	3×10^{-2}	1×10^{-4}	–	6×10^{-1}	5×10^{-1}	9×10^{-2}
GS Tips*	3×10^{-6}	1×10^{-11}	3×10^{-7}	2×10^{-12}	9×10^{-2}	–	5×10^{-1}	3×10^{-2}
AS Tips*	1×10^{-1}	1×10^{-2}	9×10^{-2}	6×10^{-2}	3×10^{-1}	4×10^{-3}	–	1×10^{-1}
Roots	1×10^{-1}	4×10^{-3}	2×10^{-2}	1×10^{-1}	9×10^{-2}	1×10^{-3}	4×10^{-1}	–

Table of *p*-values for pairwise KDE testing on plant and animal groups comparing feature spaces. The lower diagonal corresponds to scale factors associated with the central means of asymmetric branching ($\bar{\beta}, \bar{\gamma}$) while the upper diagonal corresponds to scale factors associated with variation in asymmetric branching ($\Delta\beta, \Delta\gamma$). Significant classification is defined as occurring when $p < 0.05$. Datasets with multiple individuals that are aggregated are indicated with asterisks (*). Of note is the enhanced differentiation that occurs when using the average scale factors as the feature space– in particular for the Mouse lung, Balsa, Piñon, Ponderosas, and GS Tips. Interestingly, the Roots exhibit greater differentiation between all other groups using the difference scale factors as the feature space.

Table S4 | Results of pairwise classification of Ponderosa individuals using scale factors that are related to hydraulics, and fluid transport ($\bar{\beta}, \Delta\beta$) and that are associated with strictly symmetric branching (β, γ) or asymmetric branching.

ASYM / SYM	POND03	POND05	POND06	POND07	POND16
POND03	–	5×10^{-2}	5×10^{-1}	7×10^{-1}	3×10^{-1}
POND05	5×10^{-1}	–	3×10^{-2}	8×10^{-2}	3×10^{-1}
POND06	3×10^{-1}	4×10^{-1}	–	4×10^{-1}	3×10^{-1}
POND07	6×10^{-1}	4×10^{-1}	5×10^{-1}	–	6×10^{-1}
POND16	2×10^{-1}	2×10^{-1}	2×10^{-1}	2×10^{-1}	–

Table of p-values for pairwise KDE testing on the Ponderosa individuals comparing different feature spaces. The lower diagonal corresponds to scale factors associated with strictly symmetric branching (β, γ), while the upper diagonal corresponds to scale factors associated with hydraulics and asymmetric radial branching $(\bar{\beta}, \Delta\beta)$. Significant classification is defined as occurring when $p < 0.05$. Note that no single pairwise comparison results in differentiation between Ponderosa individuals as per the $p < 0.01$ threshold.

Table S5 | Results of pairwise classification of the human head and torso individuals using scale factors that are related to hydraulics and fluid transport and that are associated with strictly symmetric branching (β, γ) or asymmetric branching ($\bar{\beta}, \Delta\beta$).

ASYM / SYM	HHT01	HHT02	HHT03	HHT04	HHT05	HHT06	HHT07	HHT08	HHT09	HHT10	HHT11	HHT12	HHT13	HHT14	HHT15	HHT16	HHT17	HHT18
HHT01	–	6×10^{-1}	7×10^{-1}	6×10^{-1}	4×10^{-1}	4×10^{-1}	3×10^{-2}	5×10^{-1}	6×10^{-1}	5×10^{-1}	1×10^{-1}	5×10^{-1}	7×10^{-1}	3×10^{-1}	2×10^{-1}	6×10^{-1}	6×10^{-1}	6×10^{-1}
HHT02	3×10^{-1}	–	7×10^{-1}	7×10^{-1}	7×10^{-1}	3×10^{-1}	6×10^{-2}	7×10^{-1}	5×10^{-1}	5×10^{-1}	5×10^{-2}	5×10^{-1}	6×10^{-1}	2×10^{-1}	2×10^{-1}	6×10^{-1}	7×10^{-1}	7×10^{-1}
HHT03	5×10^{-1}	3×10^{-1}	–	8×10^{-1}	6×10^{-1}	3×10^{-1}	5×10^{-2}	7×10^{-1}	7×10^{-1}	7×10^{-1}	1×10^{-1}	6×10^{-1}	7×10^{-1}	2×10^{-1}	4×10^{-1}	7×10^{-1}	7×10^{-1}	7×10^{-1}
HHT04	6×10^{-1}	4×10^{-1}	4×10^{-1}	–	6×10^{-1}	4×10^{-1}	3×10^{-2}	7×10^{-1}	6×10^{-1}	5×10^{-1}	5×10^{-2}	6×10^{-1}	5×10^{-1}	2×10^{-1}	3×10^{-1}	6×10^{-1}	7×10^{-1}	7×10^{-1}
HHT05	2×10^{-1}	6×10^{-1}	3×10^{-1}	4×10^{-1}	–	2×10^{-1}	9×10^{-3}	6×10^{-1}	4×10^{-1}	4×10^{-1}	7×10^{-3}	2×10^{-1}	4×10^{-1}	1×10^{-1}	3×10^{-1}	6×10^{-1}	6×10^{-1}	7×10^{-1}
HHT06	7×10^{-2}	3×10^{-2}	1×10^{-1}	6×10^{-2}	2×10^{-2}	–	5×10^{-1}	3×10^{-1}	4×10^{-1}	3×10^{-1}	6×10^{-1}	9×10^{-1}	2×10^{-1}	6×10^{-1}	2×10^{-1}	2×10^{-1}	6×10^{-1}	3×10^{-1}
HHT07	3×10^{-1}	2×10^{-2}	2×10^{-1}	2×10^{-1}	1×10^{-2}	2×10^{-1}	–	1×10^{-2}	2×10^{-1}	3×10^{-1}	6×10^{-1}	2×10^{-1}	3×10^{-3}	2×10^{-1}	3×10^{-1}	1×10^{-2}	2×10^{-1}	7×10^{-2}
HHT08	6×10^{-1}	4×10^{-1}	4×10^{-1}	6×10^{-1}	4×10^{-1}	3×10^{-2}	2×10^{-1}	–	3×10^{-1}	5×10^{-1}	5×10^{-2}	6×10^{-1}	4×10^{-1}	3×10^{-1}	1×10^{-1}	5×10^{-1}	8×10^{-1}	8×10^{-1}
HHT09	6×10^{-1}	4×10^{-1}	5×10^{-1}	6×10^{-1}	3×10^{-1}	2×10^{-1}	4×10^{-1}	5×10^{-1}	–	3×10^{-1}	4×10^{-1}	7×10^{-1}	5×10^{-1}	5×10^{-1}	6×10^{-1}	5×10^{-1}	5×10^{-1}	6×10^{-1}
HHT10	4×10^{-1}	3×10^{-1}	4×10^{-1}	3×10^{-1}	2×10^{-1}	2×10^{-1}	2×10^{-1}	4×10^{-1}	3×10^{-1}	–	2×10^{-1}	6×10^{-1}	5×10^{-1}	1×10^{-1}	3×10^{-1}	5×10^{-1}	5×10^{-1}	5×10^{-1}
HHT11	3×10^{-1}	3×10^{-2}	2×10^{-1}	1×10^{-1}	1×10^{-2}	2×10^{-1}	6×10^{-1}	2×10^{-1}	5×10^{-1}	2×10^{-1}	–	6×10^{-1}	3×10^{-2}	7×10^{-1}	4×10^{-1}	5×10^{-2}	2×10^{-1}	5×10^{-2}
HHT12	5×10^{-1}	3×10^{-1}	5×10^{-1}	5×10^{-1}	2×10^{-1}	1×10^{-1}	2×10^{-1}	5×10^{-1}	5×10^{-1}	6×10^{-1}	3×10^{-1}	–	3×10^{-1}	8×10^{-1}	3×10^{-1}	4×10^{-1}	8×10^{-1}	6×10^{-1}
HHT13	6×10^{-1}	5×10^{-1}	6×10^{-1}	7×10^{-1}	4×10^{-1}	4×10^{-2}	2×10^{-1}	6×10^{-1}	6×10^{-1}	3×10^{-1}	1×10^{-1}	4×10^{-1}	–	1×10^{-1}	2×10^{-1}	6×10^{-1}	4×10^{-1}	5×10^{-1}
HHT14	2×10^{-1}	2×10^{-2}	2×10^{-1}	1×10^{-1}	3×10^{-2}	1×10^{-1}	2×10^{-1}	2×10^{-1}	2×10^{-1}	6×10^{-1}	3×10^{-1}	5×10^{-1}	1×10^{-1}	–	4×10^{-1}	2×10^{-1}	5×10^{-1}	3×10^{-1}
HHT15	5×10^{-1}	2×10^{-1}	6×10^{-1}	4×10^{-1}	2×10^{-1}	6×10^{-2}	3×10^{-1}	5×10^{-1}	4×10^{-1}	3×10^{-1}	4×10^{-1}	5×10^{-1}	4×10^{-1}	3×10^{-1}	–	2×10^{-1}	3×10^{-1}	3×10^{-1}
HHT16	5×10^{-1}	6×10^{-1}	4×10^{-1}	6×10^{-1}	5×10^{-1}	2×10^{-2}	5×10^{-2}	5×10^{-1}	5×10^{-1}	3×10^{-1}	6×10^{-2}	3×10^{-1}	6×10^{-1}	6×10^{-2}	4×10^{-1}	–	5×10^{-1}	7×10^{-1}
HHT17	4×10^{-1}	6×10^{-1}	6×10^{-1}	5×10^{-1}	5×10^{-1}	1×10^{-1}	3×10^{-1}	6×10^{-1}	5×10^{-1}	3×10^{-1}	4×10^{-1}	5×10^{-1}	5×10^{-1}	2×10^{-1}	5×10^{-1}	5×10^{-1}	–	7×10^{-1}
HHT18	5×10^{-1}	3×10^{-1}	5×10^{-1}	4×10^{-1}	3×10^{-1}	9×10^{-2}	1×10^{-1}	5×10^{-1}	3×10^{-1}	7×10^{-1}	1×10^{-1}	5×10^{-1}	4×10^{-1}	4×10^{-1}	3×10^{-1}	4×10^{-1}	4×10^{-1}	–

Table of p-values for pairwise KDE testing on the human head and torso individuals comparing different feature spaces. The lower diagonal corresponds to scale factors that are related to hydraulics and space filling and that are associated with strictly symmetric branching (β, γ) while the upper diagonal corresponds to scale factors associated with just hydraulics and asymmetric radial branching $(\bar{\beta}, \Delta\beta)$. Significant classification is defined as occurring when $p < 0.05$. Note that only pairwise comparisons between HHT individuals 13 and 7, 11 and 5, and 7 and 5 result in differentiation between HHT individuals as per the $p < 0.01$ threshold. All other pairwise comparisons are indistinguishable.

Table S6 | Results of pairwise classification of tree tips species using scale factors associated with strictly symmetric branching (β, γ), or asymmetric branching, hydraulics, and fluid transport ($\bar{\beta}, \Delta\beta$).

SYM \ ASYM	Maple	Oak	Robinia	Whitefir	Dougfir	Whitepine
Maple	–	3×10^{-2}	7×10^{-1}	4×10^{-10}	1×10^{-2}	8×10^{-3}
Oak	4×10^{-1}	–	3×10^{-2}	9×10^{-6}	4×10^{-3}	1×10^{-1}
Robinia	5×10^{-1}	2×10^{-1}	–	3×10^{-6}	4×10^{-2}	3×10^{-2}
Whitefir	3×10^{-1}	1×10^{-1}	4×10^{-1}	–	2×10^{-1}	3×10^{-1}
Dougfir	4×10^{-1}	2×10^{-1}	5×10^{-1}	5×10^{-1}	–	1×10^{-1}
Whitepine	5×10^{-1}	3×10^{-1}	6×10^{-1}	6×10^{-1}	6×10^{-1}	–

Table of *p*-values for pairwise KDE testing on tree tips across species comparing different feature spaces. The lower diagonal corresponds to scale factors that are related to hydraulics and space filling and that are associated with strictly symmetric branching (β, γ) while the upper diagonal corresponds to scale factors associated with just hydraulics and asymmetric radial branching $(\bar{\beta}, \Delta\beta)$. Significant classification is defined as occurring when $p < 0.05$. All species had multiple individuals that were aggregated due to low branch number per individual. Of note is the clustered differentiation between Angiosperm and Gymnosperm species using the radial asymmetric scale factors, specifically the Whitefir with all Angiosperms, the Dougfir with the Oak, and the Whitepine with the Maple.

References

1. Duong T, Goud B, Schauer K. Closed-form density-based framework for automatic detection of cellular morphology changes. Proceedings of the National Academy of Sciences. 2012;109(22):8382–8387.

2. Duong T. Local significant differences from nonparametric two-sample tests. Journal of Nonparametric Statistics. 2013;25(3):635–645.

3. Dray S, Josse J. Principal component analysis with missing values: a comparative survey of methods. Plant Ecology. 2015;216(5):657–667.

4. Newberry MG, Ennis DB, Savage VM. Testing Foundations of Biological Scaling Theory Using Automated Measurements of Vascular Networks. PLoS Computational Biology. 2015;11(8):e1004455.

5. Bentley LP, Stegen JC, Savage VM, Smith DD, von Allmen EI, Sperry JS, et al. An empirical assessment of tree branching networks and implications for plant allometric scaling models. Ecology Letters. 2013;16(8):1069–1078.

6. Brummer AB, Savage VM, Enquist BJ. A general model for metabolic scaling in self-similar asymmetric networks. PLoS Computational Biology. 2017 03;13(3):1–25.

7. Hastie T, Tibshirani R, Friedman J. The Elements of Statistical Learning: Data Mining, Inference, and Prediction. 2nd ed. Springer; 2016.

8. Moses AM. Statistical Modeling and Machine Learning for Molecular Biology. CRC Press; 2017.

9. Tekin E, Hunt D, Newberry MG, Savage VM. Do Vascular Networks Branch Optimally or Randomly across Spatial Scales? PLoS Computational Biology. 2016 11;12(11):1–28.

10. Yang J, Yu L, Rennie MY, Sled JG, Henkelman RM. Comparative structural and hemodynamic analysis of vascular trees. American Journal of Physiology-Heart and Circulatory Physiology. 2010;298(4):H1249–H1259.

11. Géron A. Hands-on machine learning with Scikit-Learn and TensorFlow: concepts, tools, and techniques to build intelligent systems. O'Reilly Media, Inc.; 2017.

12. West GB, Brown JH, Enquist BJ. A general model for the origin of allometric scaling laws in biology. Science. 1997;276(5309):122–126.

13. Savage VM, Deeds EJ, Fontana W. Sizing Up Allometric Scaling Theory. PLoS Computational Biology. 2008 09;4(9):e1000171.

14. Kleiber M. Body size and metabolism. Hilgardia. 1932 January;6(11):315–353.

15. Savage VM, Gillooly J, Woodruff W, West G, Allen A, Enquist B, et al. The predominance of quarter-power scaling in biology. Functional Ecology. 2004;18(2):257–282.

16. Mori S, Yamaji K, Ishida A, Prokushkin SG, Masyagina OV, Hagihara A, et al. Mixed-power scaling of whole-plant respiration from seedlings to giant trees. Proceedings of the National Academy of Sciences. 2010;107(4):1447–1451.

17. Horton RE. Erosional development of streams and their drainage basins; hydrophysical approach to quantitative morphology. Geological society of America bulletin. 1945;56(3):275–370.

18. Strahler AN. Quantitative analysis of watershed geomorphology. Eos, Transactions American Geophysical Union. 1957;38(6):913–920.

19. Kolokotrones T, Savage V, Deeds EJ, Fontana W. Curvature in metabolic scaling. Nature. 2010 Apr;464(7289):753–6.
